# Engineering microbial consortia of *Elizabethkingia meningoseptica* and *Escherichia coli* strains for the biosynthesis of vitamin K2

**DOI:** 10.1186/s12934-022-01768-7

**Published:** 2022-03-12

**Authors:** Qiang Yang, Zhiming Zheng, Genhai Zhao, Li Wang, Han Wang, XiuMin Ding, Chunxu Jiang, Chu Li, Guoliang Ma, Peng Wang

**Affiliations:** 1 Institute of Intelligent Machines, Hefei Institutes of Physical Science, Chinese Academy of Sciences, Hefei, 230031 People’s Republic of China; 2 University of Science and Technology of China, Hefei, 230026 People’s Republic of China; 3 Hefei Normal University, Hefei, 230601 People’s Republic of China

**Keywords:** Vitamin K2, Microbial consortia, Metabolic engineering, *Elizabethkingia meningoseptica*

## Abstract

**Background:**

The study and application of microbial consortia are topics of interest in the fields of metabolic engineering and synthetic biology. In this study, we report the design and optimisation of *Elizabethkingia meningoseptica* and *Escherichia coli* co-culture, which bypass certain limitations found during the molecular modification of *E. meningoseptica*, such as resistance to many antibiotics and fewer available molecular tools.

**Results:**

The octaprenyl pyrophosphate synthase from *E. meningoseptica* sp. F2 (EmOPPS) was expressed, purified, and identified in the present study. Then, owing to the low vitamin K2 production by *E. coli* or *E. meningoseptica* sp. F2 monoculture, we introduced the *E. meningoseptica* and *E. coli* co-culture strategy to improve vitamin K2 biosynthesis. We achieved production titres of 32 mg/L by introducing vitamin K2 synthesis-related genes from *E. meningoseptica* sp. F2 into *E. coli*, which were approximately three-fold more than the titre achieved with *E. meningoseptica* sp. F2 monoculture. This study establishes a foundation for further engineering of MK-n (n = 4, 5, 6, 7, 8) in a co-cultivation system of *E. meningoseptica* and *E. coli*. Finally, we analysed the surface morphology, esterase activity, and membrane permeability of these microbial consortia using scanning electron microscopy, confocal laser scanning microscopy, and flow cytometry, respectively. The results showed that the co-cultured bacteria were closely linked and that lipase activity and membrane permeability improved, which may be conducive to the exchange of substances between bacteria.

**Conclusions:**

Our results demonstrated that co-culture engineering can be a useful method in the broad field of metabolic engineering of strains with restricted molecular modifications.

**Supplementary Information:**

The online version contains supplementary material available at 10.1186/s12934-022-01768-7.

## Background

Isoprenylated quinones, in which the length of the isoprenoid side chain varies, are widely distributed in almost all living organisms [1]. One isoprenylated quinone of particular interest is vitamin K2, a well-known high-value product. Vitamin K2 (menaquinone) is a series of vitamers with multiple isoprene units at the 3′ position of the naphthoquinone ring structure. Menaquinones have variable side chain lengths of 4*–*13 isoprene units, referred to as MK-n, where M stands for menaquinone, K stands for vitamin K, and n denotes the number of isoprenoid residues [2, 3]. Vitamin K is necessary for the functional modification of proteins that are involved in hepatic blood anticoagulation, the maintenance of bone health and cardiovascular health, cancer prevention, inflammation suppression, prevention of oxidative damage to the brain, sphingolipid synthesis, osteoporosis prevention, and even treatment for mitochondrial pathologies, such as Parkinson’s disease and amyotrophic lateral sclerosis [4–8]. To date, some of the widely examined vitamin K2-producing microorganisms include *Bacillus subtilis natto*, *B. subtilis 168*, *Elizabethkingia meningoseptica*, and *Escherichia coli*. Among them, naphthoquinone-type menaquinone-4 (MK-4), MK-5, and MK-6 have been previously identified in *E. meningoseptica* [9, 10]. *E. coli* primarily produces a benzoquinone-type ubiquinone-8 (UQ-8) under aerobic conditions but mainly synthesises MK-8 under anaerobic conditions [11].

To date, isopentenyl pyrophosphate (IPP) and its isomer dimethylallyl pyrophosphate (DMAPP), which is the precursor with variable side chain lengths, can be produced mainly via two different pathways: the methylerythritol 4-phosphate (MEP) pathway and the mevalonate (MVA) pathway [12]. Although stoichiometric studies have revealed that the 1-deoxy-d-xylulose-5-phosphate (DXP) pathway is more efficient than the MVA pathway, the production of isoprenoids by optimising the DXP pathway cannot go beyond the levels accomplished by expressing a heterologous MVA pathway in prokaryotes [13–15].

We sequenced the *E. meningoseptica* sp*.* F2 genome, and the results of KEGG pathway analysis revealed that *E. meningoseptica* sp*.* F2 utilises the MVA pathway (Additional file 1: Fig. S1) [16]. Previous studies have shown that triggering the MVA pathway could increase the level of terpenoid synthesis [17, 18]. However, there are only a few reports on the molecular modification of *E. meningoseptica*, and the main problems are listed as follows: (1) *E. meningoseptica* is resistant to multiple antibiotics, such as extended-spectrum β-lactam agents and aminoglycosides [19, 20], (2) *E. meningoseptica* lacks available molecular tools for molecular manipulation. Compared with other expression systems, *E. coli* remains the preferred host for producing recombinant proteins because of several advantages, including rapid, inexpensive, and high-yield protein production, due to the well-characterised genetics and variety of available molecular tools [21]. Several studies have explored the benefits of developing microbial consortia based on the engineering of microbial chassis [22–25]. Two *E. coli* strains or *E. coli* strains co-cultured with other strains were constructed individually to accommodate different pathway modules, which helped reduce the overwhelming metabolic stress on each strain [26, 27]. Therefore, the microbial consortium (engineered *E. meningoseptica* and *E. coli*) may be a better strategy to solve the aforementioned problems.

In this study, vitamin K2 was produced by the *E. meningoseptica* and *E. coli* co-culture system by introducing the MVA pathway and prenyltransferase genes from *E. meningoseptica* sp. F2 into *E. coli*. Compared to a single strain, the co-culture system performed effectively in the production of vitamin K2. To the best of our knowledge, this is the first study to report the usefulness of a co-culture system for vitamin K2 production.

## Results and discussion

### Engineering *E. meningoseptica* and *E. coli* co-culture systems to produce MK-n

Based on our previous antibiotic susceptibility tests, *E. meningoseptica* sp. F2 was resistant to ampicillin (0–200 µg/mL), caramycin (0–200 µg/mL), tetracycline (0–150 µg/mL), chloramphenicol (0–150 µg/mL), gentamicin (0–200 µg/mL), and streptomycin (0–200 µg/mL). These results also revealed that *E. meningoseptica* sp. F2 was sensitive to chloramphenicol (approximately 100–150 µg/mL) and tetracycline (approximately 75–150 µg/mL) [16]. To the best of our knowledge, fewer molecular modification tools for *E. meningoseptica* have been reported; therefore, performing molecular modifications on *E. meningoseptica* sp. F2 directly may be difficult*.* Considering that *E. coli* has many advantages from quick growth to high cell densities, a wide range of genetic tools and many expression vectors have been commercialised. More importantly, several researchers successfully used an *E. coli* co-culture system to improve target products [26]. In addition, *E. coli* has a complete vitamin K2 synthesis pathway (KEGG pathway: eco00130). Thus, we selected the method of co-cultivation of *E. meningoseptica* and *E. coli* to overcome these problems and improve vitamin K2 biosynthesis.

**Fig. 1:**
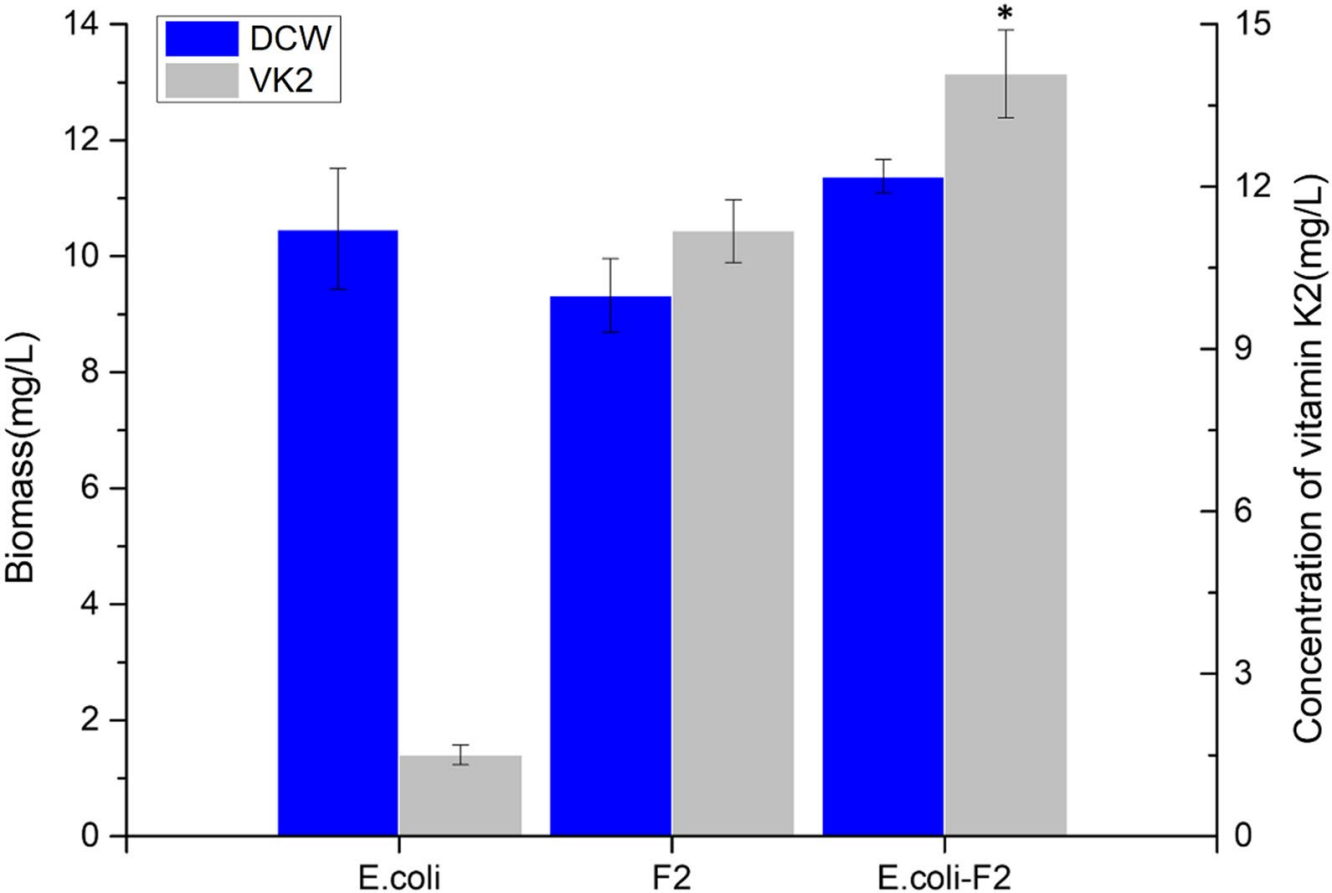
Production of vitamin K2 by the monoculture and co-culture systems of *Escherichia coli* and *Elizabethkingia meningoseptica* sp. F2. The error bars represent the standard error of at least three biological replicates. *Statistical significance (*p* < 0.05) compared to the monoculture system of *E. coli* and *E. meningoseptica* sp. F2

The products of MK-n (n = 4, 5, 6, 7, 8) were detected by co-culture of *E. meningoseptica* and *E. coli* (Additional file 1: Fig. S2). Previously, we identified that *E. meningoseptica* can produce MK-4, MK-5, and MK-6 and that *E. coli* can produce MK-8 [28, 29]. As shown in Fig. 1, the MK-n (n = 4, 5, 6, 7, 8) production and cell concentration [dry cell weight (DCW)] were measured as 14.08 ± 0.81 mg/L and 11.37 ± 0.28 mg/L, respectively. Among them, the MK series compounds were identified as MK-6 (approximately 75%) and MK-7 (approximately 10%). The results of the KEGG pathway analysis showed that *E. meningoseptica* sp. F2 did not contain either hexaprenyl diphosphate synthase (C30) or heptaprenyl diphosphate synthase (C35). The results of the KEGG pathway analysis revealed that geranylgeranyl diphosphate synthase (EmGGPPS) [EC: 2.5.1.12.5.1.10.2.5.1.29] and octaprenyl pyrophosphate synthase (EmOPPS) [EC: 2.5.1.90] existed in *E. meningoseptica* sp. F2 (Additional file 1: Fig. S1). In a previous study, we identified that EmGGPPS can catalyse consecutive condensation reactions of substrate molecules to form a C20 short-chain product GGPP [30]. Nevertheless, how the synthesis of hexaprenyl diphosphate in *E. meningoseptica* occurs remains unclear*. E. meningoseptica* sp. F2 possesses MK-6 as the major menaquinone component, and the results of the KEGG pathway analysis did not show the presence of hexaprenyl diphosphate synthase (HexppS). Therefore, we speculate that the biosynthesis of MK-6 may be related to EmOPPS in *E. meningoseptica* sp. F2. We also analysed the enzyme activity of EmOPPS derived from *E. meningoseptica* sp. F2.

### Expression and purification of a recombinant version of EmOPPS

**Fig. 2:**
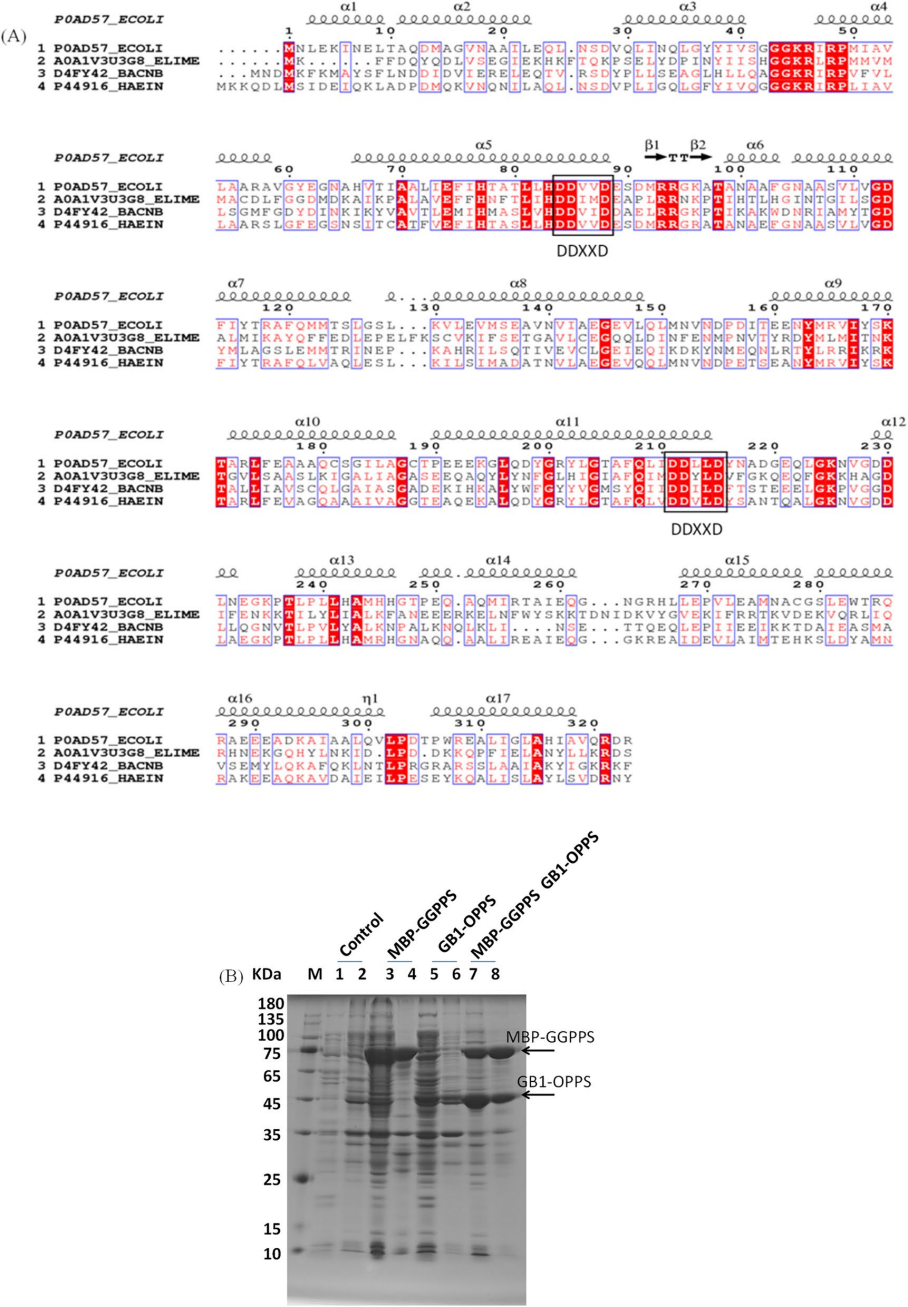
Bioinformatic and SDS-PAGE analysis of EmOPPS. **A** Alignment of amino acid sequences of prenyltransferases. The black box on a colour background indicates similar amino acid residues. The two conserved DD*XX*D motifs are indicated. **B** SDS-PAGE analysis of recombinant protein levels in *Escherichia coli* BL21 (DE3) cells at 30 °C for 4 h. Lanes 1, 3, 5, 7: supernatant. Lanes 2, 4, 6, 8: precipitate

To further investigate the significance of EmOPPS in *E. meningoseptica* sp. F2, the amino acid sequences of several trans-prenyltransferases, including C40-OPPS from *E. coli* K-12 MG1655 (P0AD57_Ecoli), *E. meningoseptica* sp. F2 (A0A1V3U058_ELIME), and *Haemophilus influenzae* Rd KW20 (P44916_HAEIN) and C35-HexppS from *B. subtilis natto* BEST195 (D4FY42_BACNB), were aligned with Clustal X and showed using ESPript. The results are shown in Fig. 2A. Two DD*XX*D motifs in the amino acid sequence were found in trans-prenyltransferases. The first motif is responsible for binding with farnesyl diphosphate (FPP), and the second with isopentenyl diphosphate (IPP) [31–33]. The amino acid located in the fifth position before the first DD*XX*D is alanine in the *E. meningoseptica* OPPS, *E. coli* OPPS, *H. influenzae* OPPS, and *B. subtilis* Hexpps and is important in determining the product’s chain length [34].

We constructed the plasmid PETA-1 (pET-28a-GB1-Em*OPPS*) and expressed it in *E. coli* BL21 to produce the strain J01. PETD-1 (pETDuet-1-MBP-Em*GGPPS*) was successfully expressed. To supplement the precursor of the side chain, we also synthesised GB1-*OPPS* on the second multiple cloning site of pETDuet-1 to form PETD-2 and transformed it into *E. coli* BL21, yielding strain J02, which can produce GB1-EmOPPS and MBP-EmGGPPS. The cells were induced with a low concentration of isopropylβ-D-1- thiogalactopyranoside (IPTG, 50 µM) and incubated at 30 °C for 4 h. The molecular masses of GB1-OPPS and MBP-GGPPS were 45.9 kDa and 80.4 kDa (Fig. 2B), respectively. To further analyse GB1-EmOPPS, we purified the proteins (Additional file 1: Fig. S3A). GB1-OPPS was purified using Ni-nitriloacetic acid (Ni–NTA) resin using an internal 6 × histidine affinity tag. Purified GB1-EmOPPS proteins were confirmed by mass spectrometry (Additional file 1: Fig. S3B).

### Enzymatic assay to detect EmOPPS activity and MK-n production in *E. coli*

To functionally characterise EmOPPS, affinity-purified His-tagged recombinant proteins were incubated with IPP and FPP as co-substrates. After the reaction, we hydrolysed the diphosphate product using phosphatases to convert it into the corresponding alcohol, which was then detected using liquid chromatography-mass spectrometry (LC–MS). As shown in Additional file 1: Fig. S4A and B, two products [C_25_H_42_O (*m/z* = 358) and C_30_H_50_O (*m/z* = 426)] were detected (Additional file 1: Table S1). This suggests that C25 and C30 are the products of EmOPPS with IPP and FPP as substrates. This is different from previously characterised OPPS, which catalyse consecutive condensation reactions of FPP with five molecules of IPP to generate C40 octaprenyl diphosphate [35, 36]. To further test the enzymatic activity of EmOPPS in vivo, we utilised the vitamin K2 biosynthesis system in *E. coli*. Previous research has shown that *E. coli* mainly synthesises one type of vitamin K2, menaquinone-8 (MK-8), under micro-anaerobic conditions [29]. MK-7 can also be produced by heterogeneous heptaprenyl pyrophosphate synthetase (HepPPS) [37]. In this study, LC–MS revealed the accumulation of MK-6, -7, and -8 by strain J01 (Additional file 1: Fig. S4C, D, Fig. S5). Combining with the results of the enzyme activity test, we concluded that the biosynthesis of MK-6 (*m/z* = 581) is related to the activity of EmOPPS. Therefore, this assay demonstrated that EmOPPS synthesised hexaprenyl diphosphate (C30) both in vivo and in vitro. In the coagulation experiments, MK-4, -5, -6 exhibited high biological activity; with the increase of the number of isoprene units, the biological activity decreased [38]. Currently, most studies focus on microbial biosynthesis of MK-7, due in part to longer biological half-life and higher yield [39, 40] and less studies on MK-6. Therefore, our findings are meaningful for the study of vitamin K2.

**Fig. 3:**
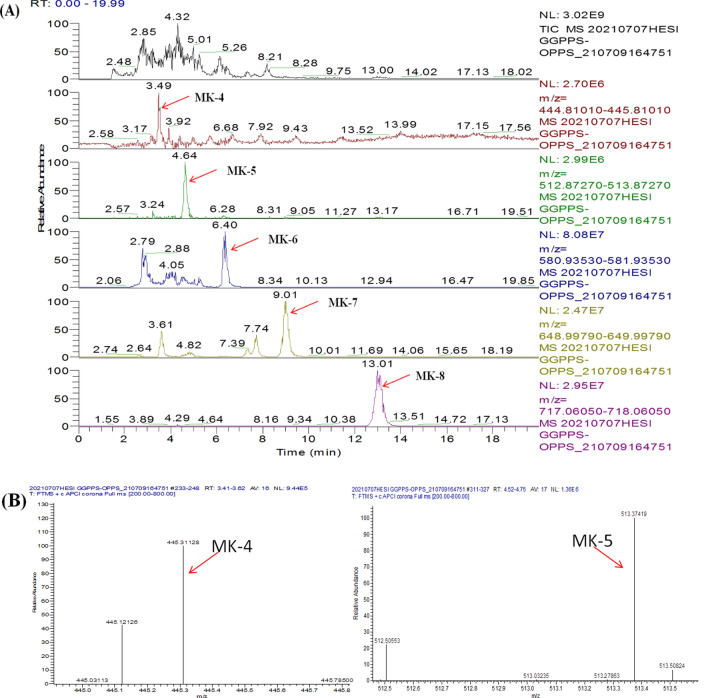

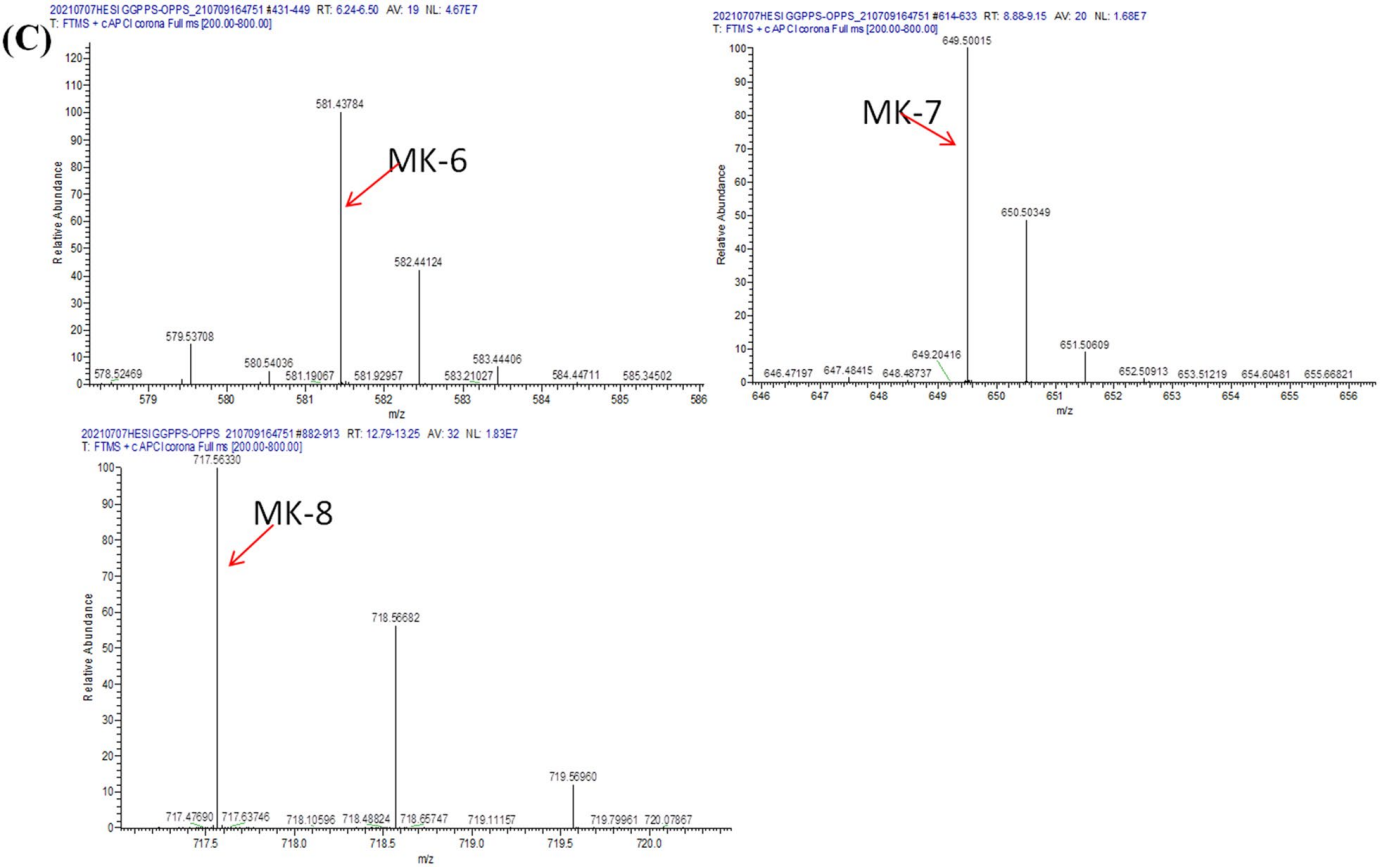
Menaquinone-n (n = 4, 5, 6, 7, 8) production in the engineered *Escherichia coli* (J02 strains). LC–MS analysis of the production of J02 strains (**A**–**C**). **A** HPLC chromatograms of different MK-n **B**, **C** mass spectra of MK-n, MK-4 (*m/z* = 445), MK-5 (*m/z* = 513), MK-6 (*m/z* = 581), MK-7 (*m/z* = 649), and MK-8 (*m/z* = 717)

The synthesis of one molecule each of C30, C35, and C40 required several molecules of IPP and one molecule of FPP. For FPP synthesis, the reaction of two molecules of IPP and one molecule of DMAPP was catalysed by GGPPS. Thus, we cloned Em*GGPPS* in pETDuet-1 to form PETD-1, and then the amplified fragments of GB1*-*EmOPPS were also cloned into the PETD-1 vector to form PETD-2. Finally, this vector was transformed into *E. coli* to form the J02 strain. This strain could biosynthesise MK-n (n = 4, 5, 6, 7, 8) (Fig. 3A, B, C, Additional file 1: Fig. S5, Additional file 1: Table S1). In a previous study, we confirmed that GGPPS can catalyse IPP and DMAPP to produce FPP and synthesise MK-4 in *E. coli* [30]. Thus, the appearance of MK-4 in the LC–MS results was related to the expression of EmGGPPS. This is the first time that a series of vitamin K2 (MK-n, n = 4, 5, 6, 7, 8) was synthesised simultaneously in *E. coli*.

**Fig. 4:**
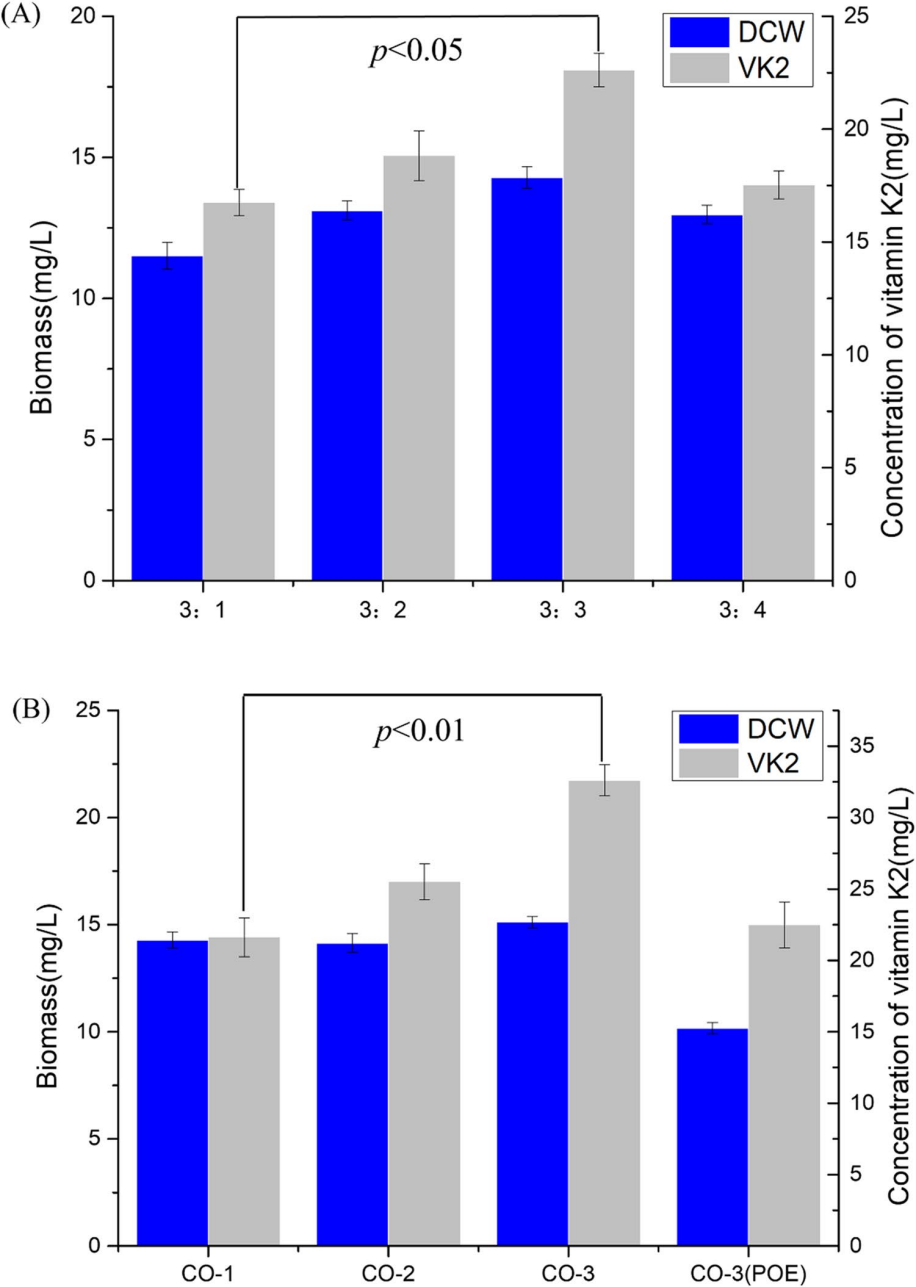
Co-culture compatibility determination and upstream strain optimisation. **A** Optimisation of the inoculation ratios for the CO-1 cultivation system. *Elizabethkingia meningoseptica* and *Escherichia coli* were inoculated into the co-culture system at a ratio of 3:1, 3:2, 3:3, and 3:4 v/v. Statistical significance (*p* < 0.05) was determined by comparing with the initial inoculation ratio (3:1). **B** Different types of co-cultivation systems. The co-culture strains were inoculated at a ratio of 3:3. The error bars represent the standard error of at least three biological replicates. Statistical significance (*p* < 0.01) compared to the CO-1 cultivation system

Co-cultures of *E. meningoseptica* sp. F2 and *E. coli* J02 (CO1 system) were investigated. This consortium, with a 3:1 inoculation ratio, was able to produce a titre of 16.74 ± 0.58 mg/L of vitamin K2 (Fig. 4A). In addition, a previous study has shown that the inoculation ratio of different strains has a greater influence on the co-cultivation system for the target product [41]. The variation in population ratio changes population dynamics, which leads to the optimal function of each pathway module for the efficient conversion of substrates to the products with little to no accumulation of intermediate metabolites [42]. Therefore, in this study, we optimised the inoculation ratio of *E. meningoseptica* sp. F2 and *E. coli*. The highest vitamin K2 production measured was 22.62 ± 0.75 mg/L and was achieved with the inoculation ratio of 3:3 (Fig. 4A). The vitamin K2 concentration was two-fold higher than that of the monoculture of *E. meningoseptica* sp. F2.

### Identification of the mevalonate pathway of *E. meningoseptica* and the enhancement of vitamin K2 production by mevalonate supply from *E. coli*

Endogenous IPP and DMAPP levels are relatively low in *E. coli* cells and are not sufficient for the high expression of heterogeneous terpenoids [24, 26]. Previous study have also shown that the production of isoprenoids by modulating the DXP pathway cannot surpass the levels achieved by expressing a heterologous MVA pathway in prokaryotes [27]. In contrast, the MVA pathway for isoprenoid biosynthesis has been frequently engineered to produce valuable compounds [28]. To increase IPP supply, we introduced the MVA pathway genes in *E. meningoseptica* sp. F2 into *E. coli*. Bioinformatic analysis revealed that *E. meningoseptica* sp. F2 uses the MVA pathway to produce isoprene precursors; no studies have previously identified and analysed this pathway. Therefore, we introduced the MVA pathway genes of *E. meningoseptica* sp. F2 into *E. coli* and identified their products. First, the four genes Em*HMGR*, Em*HMGS*, Em*PVD*, and Em*IDI*, derived from *E. meningoseptica* sp. F2, as well as Sce*MK* and Sce*PMK* derived from *Saccharomyces cerevisiae,* were constructed into two plasmids: PETA-2 and pACD2. They were then introduced into the J02 strain to form the strain H01. Subsequently, we identified the mevalonate product of H01 strains, and the mass spectrometry results showed that the engineered strain H01 could metabolise and produce mevalonate and secrete it outside the cell (Additional file 1: Fig. S6). Co-cultures of *E. meningoseptica* sp. F2 and *E. coli* H01 (CO2 system) produced 25.51 ± 1.25 mg/L of MK-n (Fig. 4B).

**Fig. 5:**
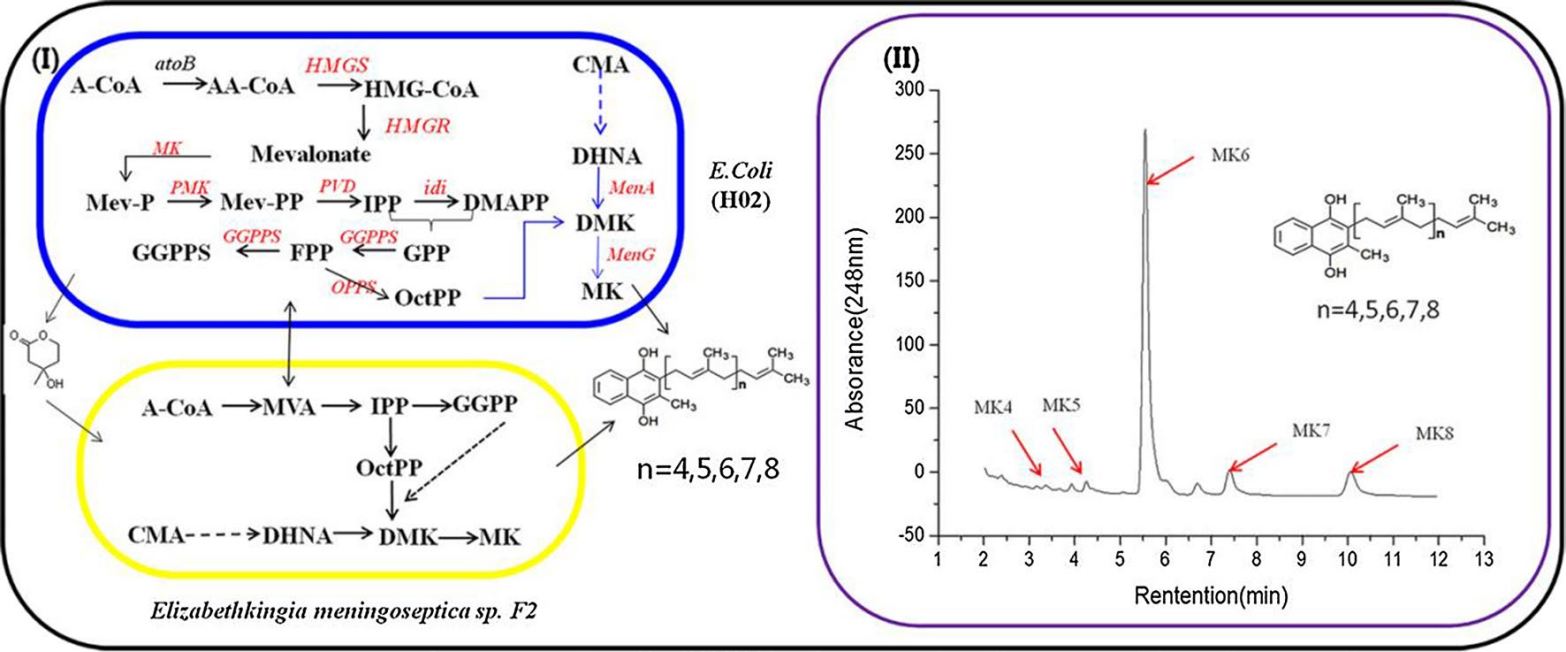
Design of *Elizabethkingia meningoseptica* and *Escherichia coli* co-culture system for vitamin K2 biosynthesis. (I) Mevalonate pathway: *atoB* acetoacetyl-CoA acetyltransferase; *HMGS* HMG-CoA synthase; *HMGR* HMG-CoA reductase; *MK* mevalonate kinase; *MVD1* mevalonate pyrophosphate decarboxylase; *idi* IPP isomerase. MenA (1,4-dihydroxy-2-naphthoate octaprenyltransferase), MenG, and UbiE (Q-8/MK-8 biosynthesis methyltransferase) encoded the head structure biosynthesis enzymes that are involved in the vitamin K2 biosynthetic pathway starting from isochorismate. Gene names in red represent the overexpressed genes. (II) HPLC analysis of vitamin K2 products in *E. meningoseptica* and *E. coli* co-culture system

The structure of menaquinone consists of a naphthoquinone ring and an isoprene side chain. The naphthoquinone ring is biosynthesised by seven enzymes encoded by the menFDHCEBA genes [39]. MenA encoded 1,4-dihydroxy-2-naphthoate octaprenyl transferase, which catalysed 1,4-dihydroxy-2- naphthoate to demethylmenaquinone as a rate-limiting enzyme. MenG/UbiE (MK-n biosynthesis methyltransferase) converted demethylmenaquinone to menaquinone. A previous study has shown that the overexpression of MenA and UbiE/MenG could be effective in improving vitamin K2 production [43]. Here, the gene fragments from Ec*MenA* derived from *E. coli K12* and Em*MenE* derived from *E. meningoseptica* sp. F2 were constructed into the plasmid PETA-2 to synthesise PETA-3. PETA-3 with pACD-2 was introduced into strain J02 to produce strain H02. When MenA or MenE/G was overexpressed, co-cultures of *E. meningoseptica* sp. F2 and *E. coli* H02 (CO3 system) produced 32.62 ± 1.1 mg/L of MK-n (n = 4, 5, 6, 7, 8), which was three-fold higher than that in the monoculture system of *E. meningoseptica* sp. F2 (Fig. 4B). As shown in Additional file 1: Fig. S7, under the same culture conditions, the yield of the *E. coli* H02 monoculture was 4.5 ± 0.13 mg/L, and the yield of the *E. meningoseptica* sp. F2 monoculture was 11.37 ± 0.28 mg/L. We speculate that strain *E. meningoseptica* sp. F2 was the dominant vitamin K2 producer in this co-culture system. These results support the further increase in vitamin K2 production using the microbial consortia platform of *E. meningoseptica* and *E. coli*. Figure 5 shows the co-culture design of *E. meningoseptica* sp. F2 and *E. coli* H02 (CO3 system) for the biosynthesis of MK-n (n = 4, 5, 6, 7, 8). The intracellular production of mevalonate by *E. coli* can be detected in the fermentation medium [44]. MVA can also be transported inside the cell as a precursor for the biosynthesis of downstream isoprenoids [15]. In addition, a previous study also indicated that the MVA feeding strategy could be more extensively utilised in the biosynthesis of isoprenoids [45]. Vitamin K2 is also an important isoprenoid product [46]; its isopentenyl side chain requires the MVA pathway to provide a precursor. In addition, both *E. coli* H02 and *E. meningoseptica* sp. F2 independently produced MK-n (n = 4, 5, 6, 7, 8). Furthermore, our previous study also implied that adding 1% polyoxyethyleneoleyl (POE) can accelerate the growth of microorganisms and improve the target product yield during the *E. meningoseptica* fermentation process [47]. Therefore, we added 1% surfactant to the co-culture system to increase the production of vitamin K2. Unexpectedly, the yield of MK-n decreased when 1% POE was added to the medium (Fig. 4B).

### The characterisation of the microbial consortium morphology

**Fig. 6:**
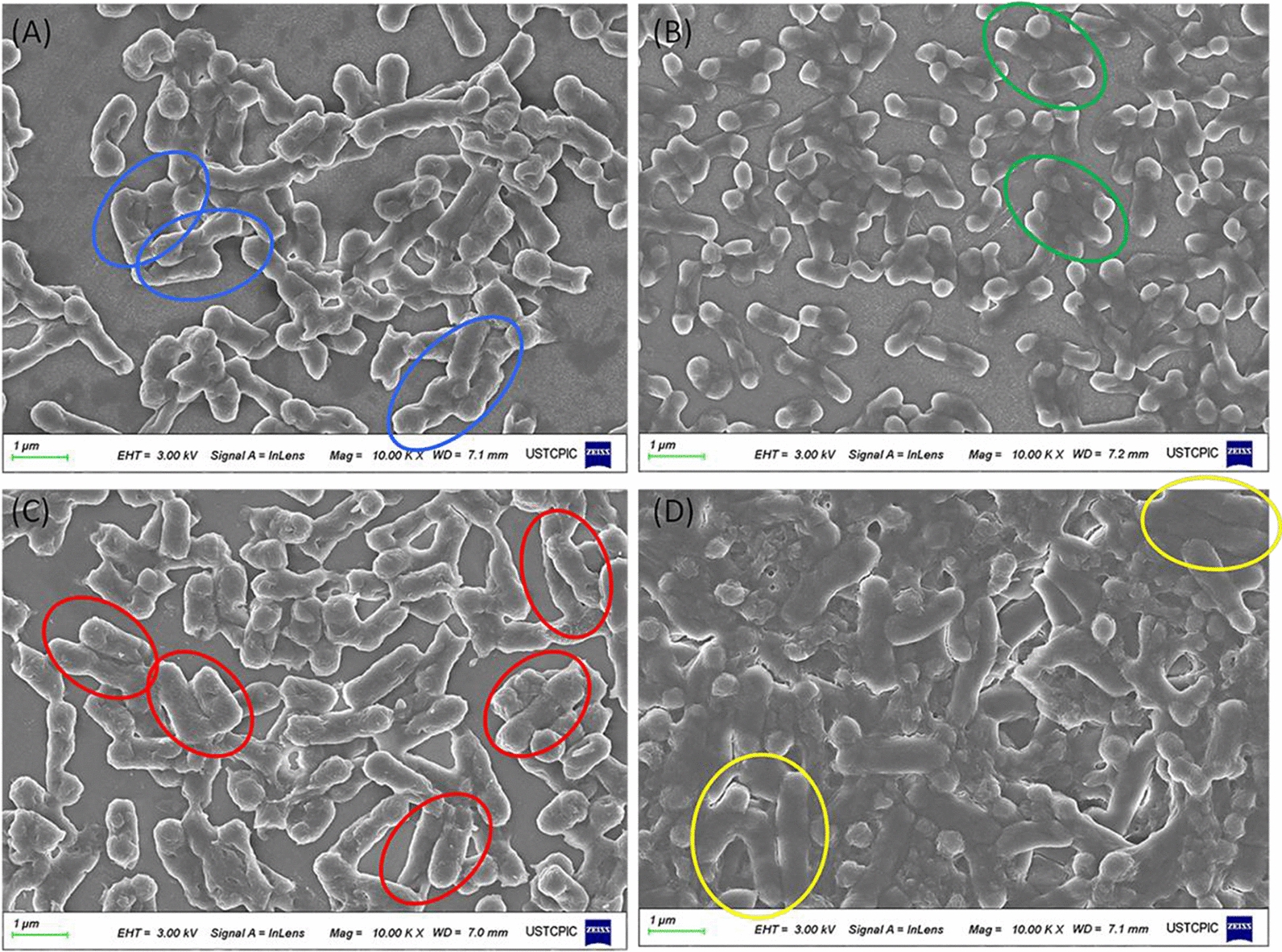
Visualisation of different inoculates using scanning electron microscopy images. **A** *Escherichia coli*. Blue circles surround the connected *E. coli* cells in view. **B** *Elizabethkingia meningoseptica* sp. F2. Green circles surround the connected *E. meningoseptica* sp. F2 cells in view. **C** *E. meningoseptica* and *E. coli* co-culture. Red circles surround the connected *E. meningoseptica* and *E. coli* cells in view. **D** *E. meningoseptica* and *E. coli* co-culture treated with 1% POE. Yellow circles surround the connected *E. meningoseptica* and *E. coli* cells in view

To investigate the presence of *E. coli* and *E. meningoseptica* sp. F2, particularly the co-existence of the microbes, the four groups (*E. coli*, *E. meningoseptica*, co-culture of *E. meningoseptica* and *E. coli*, and co-culture of *E. meningoseptica* and *E. coli* (+ POE) were visualised using scanning electron microscopy and confocal laser scanning microscopy, as shown in Fig. 6 and Additional file 1: Fig. S8. The scanning electron microscopy (SEM) images of the samples showed that *E. meningoseptica* and *E. coli* cells co-existed in the cultivation medium and that the two were closely connected. The monocultures of *E.coli* or *E. meningoseptica* sp. F2 are shown in Fig. 6A, B as controls, respectively. The size of the adjacent bacteria was uniform, and *E. coli* was partially autolysed and ruptured (Fig. 6A). *E. coli* cells were approximately 2 μm long and appeared as single straight rod bacteria. In another group, the size of the adjacent cells was generally uniform, and the shape of the cells was smooth but partially depressed (Fig. 6B). The cell volumes were smaller than those of *E. coli* (Additional file 1: Fig. S8A, B). The cells were non-spore-forming rods of approximately 1 μm in length. The co-existence of *E. meningoseptica* and *E. coli* displayed closely connected associations of large and small bacteria in a microbial consortium (Fig. 6C, Additional file 1: Fig. S8C, D). *E. coli* are gram-negative bacteria, with short rods, 2.0–6.0 μm in length; *E. meningoseptica* sp. F2 are gram-negative bacteria, with non-motile, non-spore-forming rods (1.0–2.0 μm length) [48–51]. Therefore, it can be seen that the longer bacteria is *E. coli*, and the other closely connected bacteria is *E. meningoseptica* sp. F2 (Fig. 6C, Additional file 1: Fig. S8C, D). The members of a consortium communicate by exchanging metabolites or signals that allow them to coordinate their activities through the division of labour [52]. Here, we speculate that the closely connected combination of bacteria may be more conducive to the exchange of materials and energy between different bacteria. Microbial interactions usually can be exploited to improve cell viability and productivity. Mutualistic interactions are established between the used strains; therefore, a relationship can benefit the interacting strains, thus promoting the overall performance [22, 53]. Furthermore, the number of cells of strain *E. meningoseptica* sp. F2 was far more than that of strain *E.coli* (Additional file 1: Fig. S8C and D). Confocal laser scanning microscopic observation also confirmed that strain *E. meningoseptica* sp. F2 was the dominant strain in this co-culture system. The yield of monoculture of *E. meningoseptica* sp. F2 was higher than that of monoculture of *E. coli*; therefore, we speculate that *E. meningoseptica* sp. F2 is the main vitamin K2-producing strain in this system. However, remarkable morphological changes were observed after the cells were subjected to POE treatment (Fig. 6D). Only a small part of the combined bacterial form was different in size and connection. In addition, there were several autolysed and ruptured bacterial fragments between the bacteria.

### Esterase activity and membrane permeability

**Fig. 7:**
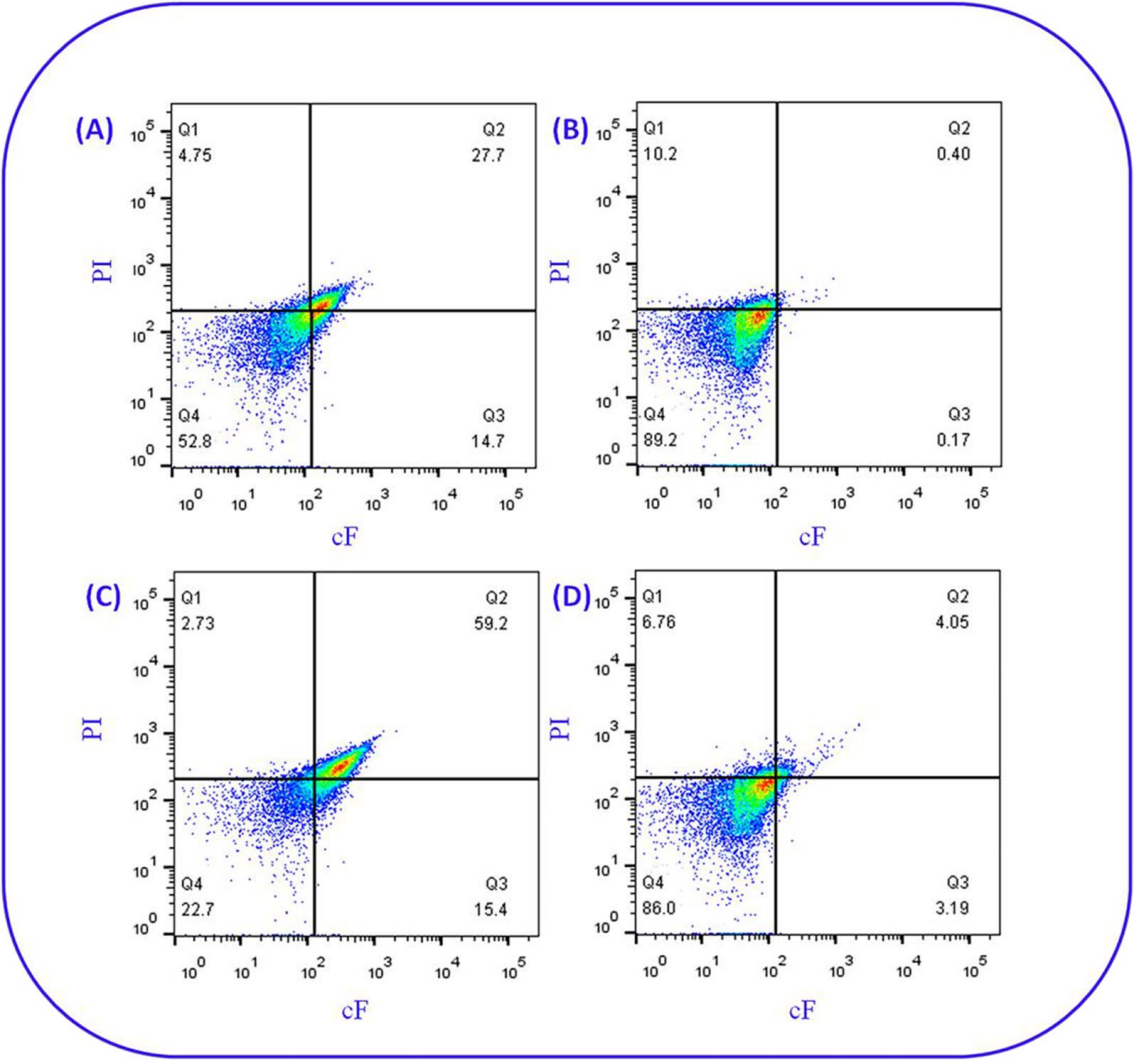
Flow cytometry dot plots of monoculture system of *Escherichia coli *(**A**), **B** and *Elizabethkingia meningoseptica* sp. F2 as a reference and **C** co-culture strains of *E. meningoseptica* and *E. coli* cells treated without or **D** with 1% POE

Flow cytometry (FCM) was used to determine the cell permeability and esterase activity of the *E. meningoseptica* and *E. coli* co-culture. Dual parameter dot plots of *E. coli* BL21, *E. meningoseptica* sp. F2, CO3, and CO3 + POE cells were stained with carboxyfluorescein diacetate (cFDA) and propidium iodide (PI), as shown in Fig. 7. The subpopulations were identified based on their differential staining characteristics with PI and carboxyfluorescein (cF): PI-stained cells (cF-negative, PI-positive; Q1 quadrant) exhibited inactivated esterase and damaged membranes; PI and cF double-stained cells (cF-positive, PI-positive; Q2 quadrant) exhibited sublethal injuries with residual esterase activity and compromised membranes; cF-stained cells (cF-positive, PI-negative; Q3 quadrant) exhibited high esterase activity and intact membranes; the unstained area (cF-negative, PI-negative; Q4 quadrant) most likely corresponded to debris or lysed cells attributable to bacterial autolysis [54, 55].

Approximately, 52.8% and 89.2% of *E. coli* and *E. meningoseptica* sp. F2 cells in the control group, respectively, were located in the Q4 quadrant (Fig. 7A, B), indicating that debris or lysed cells were attributable to bacterial autolysis during this period. Moreover, we observed 59.2% double-stained cells (cF-positive, PI-positive; Q2 quadrant) in Fig. 7C, which indicated that they exhibited higher esterase activity and membrane permeability in the co-culture of *E. meningoseptica* and *E. coli.* When compared with other groups (Fig. 7A, B, D), a minor proportion was distinguished in the Q1 quadrant (2.73%) (cells presumably without metabolic activity) and Q4 quadrant (22.7%) (most likely corresponded to debris or lysed cells attributable to bacterial autolysis). Therefore, we speculate that the construction of a microbial consortium of *E. meningoseptica* and *E. coli* improves membrane permeability and may be beneficial for the exchange and metabolism of substances between bacteria. Such microbial consortia consist of several organisms that, together, are more stable to environmental challenges, display reduced metabolic burden due to a division of labour and exchange of resources, possess expanded metabolic capabilities relative to monocultures, and can communicate (chemically or physically) between strains [56–58]. However, different phenomena were observed with initial concentrations of 1% POE, with most cells of CO3 located in the Q4 quadrant (Fig. 7D). The results revealed that most of the cell membranes of the CO3 co-cultivation system cells were damaged by exposure to 1% POE. According to a previous study, surfactants have been added to the culture to improve production; low concentrations of surfactants have been reported to increase membrane permeability; therefore, extracellular productivity was promoted by restricting intracellular diffusion of target metabolites [59]. Combined with the electron microscopy results in Fig. 6, we found that several cells were autolysed and lysed. Previous studies have found that a single *E. meningoseptica* sp. F2 can tolerate a concentration of 1% POE and not die. We speculate that, in this mixed bacterial system, the death of *E. coli* may be accelerated by POE.

## Conclusion

To the best of our knowledge, this is the first report on the use of a bacterial co-culture system for the synthesis of vitamin K2. The present study showed that co-culture of *E. meningoseptica* and *E. coli* could be used to improve MK-n (n = 4, 5, 6, 7, 8) production by introducing vitamin K2 synthesis pathway genes from *E. meningoseptica* sp. F2 in *E. coli*. To investigate the molecular mechanism of MK-n production in *E. meningoseptica*, an enzymatic assay was performed, which revealed that EmOPPS could catalyse consecutive condensation reactions of FPP with three molecules of IPP to generate C30 HexPP in vivo and in vitro, which constitutes the side chain of MK-6. In addition, MK-n (n = 4, 5, 6, 7, 8) production was initially achieved in engineered *E. coli* by overexpressing *E. meningoseptica* sp. F2-derived EmOPPS and EmGGPPS. Under the optimised inoculation ratio and overexpression of synthetic pathway genes, MK-n (n = 4, 5, 6, 7, 8) production significantly increased to 32.62 ± 1.1 mg/L, which was approximately three-fold higher than that in the *E. meningoseptica* sp. F2 monoculture. Further expansion of this co-culture, morphological analysis, and FCM illustrated the physiological state of bacteria under co-culture conditions. The twin-bacillus consortium of *E. meningoseptica* and *E. coli* that we developed might serve as a promising platform for improved vitamin K2 biosynthesis.

## Materials and methods

### Bacterial strains, media, and cultivation

*E. coli* DH5α and *E. coli* BL21 (DE3) were used as host strains for plasmid construction and protein expression, respectively. Luria–Bertani (LB) medium (10 g/L tryptone, 5 g/L yeast extract, and 10 g/L NaCl) was used to grow *E. coli* cells unless otherwise specified. The strain *E. meningoseptica* sp. F2 is commercially used for industrial vitamin K2 production (China Centre for Type Culture Collection, CCTCC, AB2011070). The fermentation medium for the cultivation of the monoculture or co-culture systems was prepared as previously described [47]. The *E. meningoseptica* sp. F2 strain was inoculated onto beef extract peptone agar slants (3 g/L beef extract, 10 g/L peptone, 5 g/L NaCl, and 20 g/L agar; pH 7) at 37 °C for 24 h [12 h for *E. coli* BL21 (DE3)] and then stored at 4 °C. Subsequently, the strain on the agar slants was inoculated into fresh seed medium (20 g/L glycerol, 33 g/L peptone, 3 g/L K_2_HPO_4_·7H_2_O, 4.5 g/L NaCl, 0.3 g/L MgSO_4_·7H_2_O, and 1.5 g/L yeast extract; pH 7.2) in 250 mL Erlenmeyer flasks with a working volume of 50 mL, incubated at 22 °C, and 300 rpm for 24 h [12 h for *E. coli* BL21 (DE3)]. Then, the pre-inoculum was transferred to a 500 mL shake flask containing 100 mL of fermentation medium (20 g/L glycerol, 33 g/L peptone, 3 g/L K_2_HPO_4_·7H_2_O, 4.5 g/L NaCl, 0.3 g/L MgSO_4_·7H_2_O, 1.5 g/L yeast extract, pH 7.2), incubated at 37 °C, and 250 rpm for 9 days. After 9 days of fermentation, biomass accumulation was measured and estimated as DCW. In brief, 25 mL of the fermentation broth was collected and centrifuged at 15,000 rpm for 15 min, and the supernatant was discarded. The wet cells were washed with sterile distilled water and freeze-dried to a constant weight.

### Genetic manipulations

All strains and plasmids used are listed in Table 1. The primer sequences are provided in Additional file 1: Table S2. The gene encoding EmOPPS derived from *E. meningoseptica* sp. F2 was amplified by polymerase chain reaction (PCR) using genomic DNA as the template. The PCR conditions were as follows: initial denaturation at 94 °C for 5 min; denaturation at 94 °C for 30 s, annealing at 54 °C for 30 s, and extension at 72 °C for 2 min for a total of 30 cycles; and the final extension at 72 °C for 10 min. Pfu PCR MasterMix (TIANGEN, Beijing, China) was used for DNA amplification. The MBP-Em*GGPPS* from V29-MBP-Em*GGPPS* was amplified and cloned into the *Nco*I and *Bam*HI sites of the pETDuet-1 vector to form PETD-1. The amplified fragments GB1*-*Em*OPPS* were also cloned into the *Nde*I and *Kpn*I sites of a PETD-1 vector to form PETD-2 (Table 1). To construct PETA-1, GB1-Em*OPPS* was cloned into the *Nco*I and *BamH*I sites of pET28a. The four genes (Em*HMGR*, Em*HMGS*, Em*PVD*, and Em*IDI*) derived from *E. meningoseptica* sp. F2 and Sce*MK* and Sce*PMK* derived from *S. cerevisiae* were constructed into two plasmids PETA-2 and PACD-2. The amplified fragments Em*HMGR* and Em*HMGS* were also cloned into the *Nco*I and *Bam*HI sites of a pACYCDuet-1 vector to form PACD-1. Then, Sce*MK* and Em*IDI* were cloned into the *Nde*I and *Kpn*I sites of PACD-1 to form PACD-2. The amplified fragments Em*PVD* and Sce*PMK* were also cloned into the *Nco*I and *Bam*HI sites of the pET28a vector to form the vector PETA-2. Ec*MenA* genes derived from *E. coli K12* and Em*MenE* derived from *E. meningoseptica* sp. F2 were constructed on the *Xho*I and *Sal*I sites of plasmid PETA-2 to form PETA-3. The recombinant plasmids were constructed using Gibson assembly [60]. NEBuilder HiFi DNA Master Assembly Mix was purchased from New England Bio Labs (NEB, Beijing, China). Restriction enzyme digestion, transformation, and other standard molecular biology techniques were performed as described by Sambrook et al*.* [61]*.* Protein expression was induced by adding 50 μM IPTG at OD_600_ = 0.6, and the cells were incubated for 4 h at 30 °C. The plasmid mini-prep kit and DNA gel extraction kit were purchased from Sangon Biotech (Shanghai, China).

**Table 1:** Strains and plasmids that were used in this study

Strains/plasmids	Relevant characteristic(s)	Source
Strains		
* E. coli* DH5α Competent Cells	F-φ80 lac ZΔM15 Δ(lacZYA-arg F) U169 endA1 recA1 hsdR17(r k^−^,m k^+^) supE44λ- thi-1 gyrA96 relA1 phoA	Sangon Biotech
*E. coli* BL21(DE3) Competent Cells	F- ompT hsdSB(rB-mB-) gal dcm(DE3)	Sangon Biotech
*Elizabethkingia meningoseptica* sp. F_2_	The original strain was obtained from China Center for Type Culture Collection (CCTCC) AB2010137. Mutagenic strains	Our lab
J01	*E. coli* BL21 carrying PETA-1	This study
J02	*E. coli* BL21 carrying PETD-2	This study
H01	*E. coli* BL21 carrying PETA-2, PACD-2, PETD-2	This study
H02	*E. coli* BL21 carrying PETA-3, PACD-2, PETD-2	This study
H03	*E. coli* BL21 carrying pET28a-GFP, PETD-2	This study
Co-cultivation system		
CO1	Co-culture of *E. meningoseptica* sp. F2 and *E. coli* J02	This study
CO2	Co-culture of *E. meningoseptica* sp. F2 and *E. coli* H01	This study
CO3	Co-culture of *E. meningoseptica* sp. F2 and *E. coli* H02	This study
CO4	Co-culture of *E. meningoseptica* sp. F2 and *E. coli* H03	This study
Plasmids		
pET28a	T7 promoter, KanR	Novagen
pET28a-GFP	pET28a carrying GFP, KanR	Novagen
pACYCDuet-1	double T7 promoters, CmR	Novagen
pETDuet™-1	double T7 promoters, Amp	Novagen
PETD-1	pETDuet™-1 carrying MBP-Em*GGPPS*, Amp	This study
PETD-2	pETDuet™-1 carrying MBP-Em*GGPPS*-GB1Em*OPPS*, Amp	This study
PETA-1	pET28a carrying GB1-Em*OPPS*-His, Kan	This study
PETA-2	pET28a carrying Sce*PMK*-Em*PVD*, Kan	This study
PETA-3	pET28a carrying Sce*PMK*-Em*PVD*-Ec*MenA*-Em*MenE*, Kan	This study
PACD-1	pACYCDuet-1 carrying Em*HMGS*-Em*HMGR*, CmR	This study
PACD-2	pACYCDuet-1 carrying Em*HMGS*-Em*HMGR*-Sce*MK*-Em*IDI*, CmR	This study

*Elizabethkingia meningoseptica* sp. F2 was obtained from the China Center for Type Culture Collection. The ID should be CCTCC AB2010137 instead of CCTCC AB2011070 [30]

The protein purification procedure has been previously described [62]. Briefly, the cells were disrupted and purified using Ni–NTA column chromatography. The bacterial cells (containing pET-28a-GB1-Em*OPPS*) were sonicated and centrifuged at 12,000×*g* for 15 min. The supernatant was then loaded onto an Ni–NTA-Sefinose™ column (Sangon Biotech). After the column was washed with a wash buffer (20 mM Tris–HCl, 500 mM NaCl, pH 8.0) and different concentrations of imidazole, the recombinant protein was eluted with an elution buffer (250 mM imidazole, 20 mM Tris–HCl, 500 mM NaCl, pH 8.0). The purified EmOPPS was analysed using mass spectrometry by Sangon Biotech.

### EmOPPS enzymatic assay and product identification

In vitro enzyme assays were performed as previously described [63, 64]. Briefly, a 400 μL reaction system containing 400 μM IPP and 200 μM FPP (100 mM HEPES, 5 mM MgCl_2_, 10 mM KCl, pH 7.5) and 0.5 μmol/L of the purified recombinant fusion protein were incubated at 30 °C for 2 h. After inoculation, the solution was mixed with 200 μL of 0.2 mol/L Tris–HCl (pH 9.5) containing bovine intestinal alkaline phosphatase (20 mg/mL, more than 10 DEA units/mg, Sigma-Aldrich) and two units of shrimp alkaline phosphatase (1 unit/μL; TaKaRa). The reaction mixture was then incubated overnight at 30 °C to hydrolyse the diphosphate products into their corresponding alcohols. The mixture was extracted with hexane, and then three parallel samples of hydrolysate were concentrated to 100 µL in the experiment. Finally, the sample products were analysed using an LC–MS system equipped with an LTQ Orbitrap XL ETD analyser (Thermo Fisher Scientific, USA). The samples were separated using an Agilent 1200 Series HPLC system at a flow rate of 250 μL/min, and the mobile phase consisted of methanol (100%). The electrospray potential was 4.5 kV in positive electrospray ionisation (ESI) mode, and the source temperature was 275 °C. Three independent cultures were analysed for each set of experiments.

### Extraction and analysis of vitamin K2 and mevalonate

The extraction of vitamin K2 and measurement of its concentration were adapted from Wei et al. [9]. Briefly, vitamin K2 analysis was performed using high-performance liquid chromatography (HPLC, Shimadzu, Kyoto, Japan) with an eclipse plus a C18 column (Shimadzu, 250 mm × 4.6 mm ID); aliquots of 20 μL were injected manually using a loop injection valve (Shimadzu). The column temperature was maintained at 35 °C. The mobile phase consisted of methanol and dichloromethane (4:1, v/v) at a flow rate of 1 mL/min. The UV–Visible detector was operated at 248 nm for the menaquinones. The menaquinone series of compounds was determined by their absorption and mass spectra using an LC–MS system equipped with a 6200 series TOF/6500 series analyser (Agilent Technologies, Santa Clara, CA, USA).

Mevalonate was determined using an Agilent System 7820A GC equipped with a flame ionisation detector and an HP-5 column (30 m × 0.25 mm, 0.25 μm film thickness) [65, 66]. Briefly, 3 mL of fermentation broth was centrifuged at 14,000×*g* for 10 min at room temperature. The supernatant was adjusted to pH 2.0–3.0 with HCl and incubated at 45 °C for 2 h to convert mevalonate to mevalonolactone. This solution was then extracted with ethyl acetate and saturated with anhydrous Na_2_SO_4_. The ethyl acetate phase containing mevalonolactone was analysed. The column temperature profile was 70 °C for 1 min, a ramp of 20 °C/min to 150 °C with a 5 min hold, and a ramp of 30 °C/min to 300 °C with a 3 min hold. The inlet temperature was 150 °C, with a split ratio of 20:1. The retention time of mevalonolactone was confirmed using the commercial standard DL-mevalonolactone.

### Morphological analysis of microbial cells by SEM and confocal laser scanning microscopy

SEM: After 3 days of fermentation, the cells were centrifuged (6000 rpm, 5 min), washed twice with phosphate-buffered saline (PBS, pH 7.4), placed on coverslips, and dried naturally. The cells were then immersed in 2.5% glutaraldehyde for 2–4 h at 4 °C, washed three times with phosphate buffer, and subjected to gradient ethanol dehydration (30%, 50%, 70%, and 90% ethanol once and 100% ethanol twice, each for 15 min). After dehydration, the cells were metal-sprayed and observed under a scanning electron microscope (Schottky Field Emission Scanning Electron Microscope, GeminiSEM 500, USA).

Laser confocal scanning microscopy: The co-culture cells (CO4 system: co-culture of *E. meningoseptica* sp. F2 and *E. coli* H03) were diluted ten-fold with PBS (pH 7.4) and placed on a glass slide. The fluorescent images were observed using a confocal laser scanning microscope (LSM 980, ZEISS, Germany) with a 488 nm bandpass filter.

### Staining procedure and flow cytometric measurement

Double staining with cFDA (Solarbio, Shanghai, China) and PI (Sigma-Aldrich, St Louis, MO, USA) was used for FC analysis [47, 67, 68]. Briefly, non-treated stained cells and cells treated at 85 °C for 15 min and subsequently stained with cFDA or PI, respectively, were used as controls to define negative and positive histogram regions. Moreover, a non-stained and non-treated control was used to determine the autofluorescence of cells. *E. coli* BL21, *E. meningoseptica* sp. F2, CO3, and CO3 + POE cells were initially incubated with 50 μM FDA at 37 °C for 15 min to allow intracellular enzymatic conversion of cFDA to cF. After labelling, the cells were centrifuged at 12,000×*g* for 5 min at 10 °C and resuspended in 1 mL PBS buffer (pH 7.0). Then, 30 μM PI was added, and the mixture was placed in an ice bath and incubated for 10 min in the dark to allow labelling of membrane-compromised cells. Following the incubation with PI, the samples were placed on ice in the dark until analysis (maximum of 1 h).

Analysis was performed using a Gallios flow cytometer (Beckman Coulter Inc., Miami, FL, USA). The forward scatter (FS), side scatters (SS), and green (FL1) and red (FL3) fluorescence of each cell were measured, amplified, and converted into digital signals for further analysis. The FL1 of the cells stained with cFDA was collected in the FL1 channel (525 ± 15 nm), whereas the FL3 of cells labelled with PI was collected in the FL2 channel (620 ± 15 nm). The flow rates and cell concentrations of the samples were adjusted to maintain the acquisition at 200 microbial cells per second. A total of 50,000 events were recorded. The trials were replicated at least three times with three samples for each short-wave UV dose. Flow cytometry data were analysed using FlowJo version vX.0.7 (TreeStar Inc., Ashland, OR, USA).

### Bioinformatic and data analyses

Multiple sequence alignments of *E. coli K-12* MG1655 (P0AD57_Ecoli), *E. meningoseptica* sp. F2 (A0A1V3U058_ELIME), *H. influenzae* RdKW 20 (P44916_HAEIN), and C35-HexppS from *B. subtilis* subsp*. natto* BEST195 (D4FY42_BACNB) were performed using Clustal X and then analysed using ESPript 3.0. The sequences of four prenyltransferases obtained from the National Center for Biotechnology Information database: P0AD57_Ecoli (NP_417654), A0A1V3U058_ELIME (AQX05701), P44916_HAEIN (AAC22540), and D4FY42_BACNB (BAI85773). Three independent cultures were analysed for each set of experiments. All data are presented as mean ± standard error and were compared with those the respective control groups. Statistical significance was assessed by one-way ANOVA via GraphPad Prism 9 software (GraphPad Software, Inc., USA), and differences with *p* < 0.05 were designated as significant in all the cases.

## Supplementary Information

**Additional file 1: Figure S1.** Terpenoid backbone biosynthesis of *E. meningoseptica *sp. F_2_ based on KEGG pathway assignment*.* *A-CoA* acetyl coenzyme A; *AA-CoA* acetoacetyl-CoA; *HMG-CoA* hydroxymethylglutaryl-CoA; *IPP* isopentenyl diphosphate; *DMAPP* dimethylallyl diphosphate; *GPP* geranyl diphosphate; *FPP* farnesyl diphosphate; *GGPP* geranylgeranyl diphosphate; *OPP* octaprenyl diphosphate. **Figure S2.** Chromatography of MK-n (n = 4, 5, 6, 7, 8) by HPLC system. **A** The sample was extracted from monoculture system of *E. meningoseptica*, **B** the sample was extracted from coculture system of *E. meningoseptica and E. coli*. **Figure S3.** **A** The purification of GB1-OPPS. M: protein molecular weight marker; lane 1: The total cell lysate supernatant flows through the column. Lanes 2–6: wash fractions with 10, 15, 20, 25, 30 mM imidazole, respectively. Lanes 7–8: Elution fractions with 250 mM imidazole, respectively. **B** Purified GB1-*Em*OPPS proteins was confirmed by mass spectrometry. **Figure S4. **Enzymatic assay of EmOPPS in vivo and vitro. **A**, **B** LC-MS analysis of the GB1-EmOPPS products with farnesyl diphosphate (FPP) and isopentenyl diphosphate (IPP) as substrates after hydrolyzation. **C**, **D** J01 strains could accumulated MK-n (n = 6, 7, 8). LC-MS analysis of the production of this strains. **A**, **C** Horizontal graphs refer to result of HPLC and vertical graphs **B**, **D** refer to mass spectra. **Figure S5.** The comparison of the mass spectrum of the fermentation results of the J01, J02 strain with that of the MK-n (n = 4, 5, 6, 7) standard, **A** MK-4, MK-5, MK-6, and MK-7 standard, respectively, for MS analysis. **B** The mass spectrum results of monocultured *E. coli* producing MK-8 as a reference. **Figure S6.** Analysis and identification of mevalonolactone by GC-MS from fermentation supernatant. **Figure S7.** The monoculture of different *E. coli* strains as comparison. The error bars represent the standard error of at least three biological replicates. Statistical significance (*p* < 0.01) compared to the original strains. **Figure S8.** Visualization of different inocula by confocal laser scanning microscope. **A** *E. meningoseptica.* **B** *E. coli*. **C**, **D** *E. meningoseptica* and *E. coli* co-culture. Red circles surround the connected *E. meningoseptica* and* E. coli* cells in view. **C** Bright field; **D** fluorescence image merged image. **Table S1.** Masses and identities of LC/MS analyses results. **Table S2.** Oligonucleotide primers used in this study.

## Data Availability

All data generated or analysed during this study are included in this published article (and its additional information files).
