# Supplementary material for: Engineering microbial consortia of Elizabethkingia meningoseptica and Escherichia coli strains for the biosynthesis of vitamin K2
Source: Microb Cell Fact. 2022 Mar 12;21:37. doi: 10.1186/s12934-022-01768-7 (PMC8917678; doi:10.1186/s12934-022-01768-7)
Supplement: Supplementary file 1 — Additional file 1: Figure S1. Terpenoid backbone biosynthesis of E. meningoseptica sp. F2 based on KEGG pathway assignment. A-CoA acetyl coenzyme A; AA-CoA acetoacetyl-CoA; HMG-CoA hydroxymethylglutaryl-CoA; IPP isopentenyl diphosphate; DMAPP dimethylallyl diphosphate; GPP geranyl diphosphate; FPP farnesyl diphosphate; GGPP geranylgeranyl diphosphate; OPP octaprenyl diphosphate. Figure S2. Chromatography of MK-n (n = 4, 5, 6, 7, 8) by HPLC system. A The sample was extracted from monoculture system of E. meningoseptica, B the sample was extracted from coculture system of E. meningoseptica and E. coli. Figure S3. A The purification of GB1-OPPS. M: protein molecular weight marker; lane 1: The total cell lysate supernatant flows through the column. Lanes 2–6: wash fractions with 10, 15, 20, 25, 30 mM imidazole, respectively. Lanes 7–8: Elution fractions with 250 mM imidazole, respectively. B Purified GB1-EmOPPS proteins was confirmed by mass spectrometry. Figure S4. Enzymatic assay of EmOPPS in vivo and vitro. A, B LC-MS analysis of the GB1-EmOPPS products with farnesyl diphosphate (FPP) and isopentenyl diphosphate (IPP) as substrates after hydrolyzation. C, D J01 strains could accumulated MK-n (n = 6, 7, 8). LC-MS analysis of the production of this strains. A, C Horizontal graphs refer to result of HPLC and vertical graphs B, D refer to mass spectra. Figure S5. The comparison of the mass spectrum of the fermentation results of the J01, J02 strain with that of the MK-n (n = 4, 5, 6, 7) standard, A MK-4, MK-5, MK-6, and MK-7 standard, respectively, for MS analysis. B The mass spectrum results of monocultured E. coli producing MK-8 as a reference. Figure S6. Analysis and identification of mevalonolactone by GC-MS from fermentation supernatant. Figure S7. The monoculture of different E. coli strains as comparison. The error bars represent the standard error of at least three biological replicates. Statistical significance (p < 0.01) compared to the original strains. Figure [file 12934_2022_1768_MOESM1_ESM.docx]

**Supplementary materials**

Engineering microbial consortia of *Elizabethkingia meningoseptica* and *Escherichia coli* strains for the biosynthesis of vitamin K2.

Qiang Yang^1,2^, Zhiming Zheng^1,*^, Genhai Zhao^1^, Li Wang^1^, Han Wang^1,2^, XiuMin Ding^1,2^, Chunxu Jiang^1,2^, Chu Li^1,3^ Guoliang Ma^1,2^, Peng Wang^1,*^

^1^ Institute of Intelligent Machines, Hefei Institutes of Physical Science, Chinese Academy of Sciences, Hefei 230031, China/P. R. China;

^2^University of Science and Technology of China, Hefei 230026, China/P. R. China;

^3^ Hefei Normal University, Hefei 230601, PR China

*Corresponding Author: Zhiming Zheng and Peng Wang,

Tel/Fax:+86-0551-55913148,e-mail: zhengzhiming2014@163.com; pengwang@ipp.ac.cn

Figure S1.Terpenoid backbone biosynthesis of *E. meningoseptica* sp. F_2_ based on KEGG pathway assignment*.* A-CoA, Acetyl coenzyme A; AA-CoA, Acetoacetyl-CoA; HMG-CoA, Hydroxymethylglutaryl-CoA; IPP, Isopentenyl diphosphate; DMAPP, Dimethylallyl diphosphate;GPP, Geranyl diphosphate;FPP, Farnesyl diphosphate; GGPP, Geranylgeranyl diphosphate; OPP, Octaprenyl diphosphate.

**
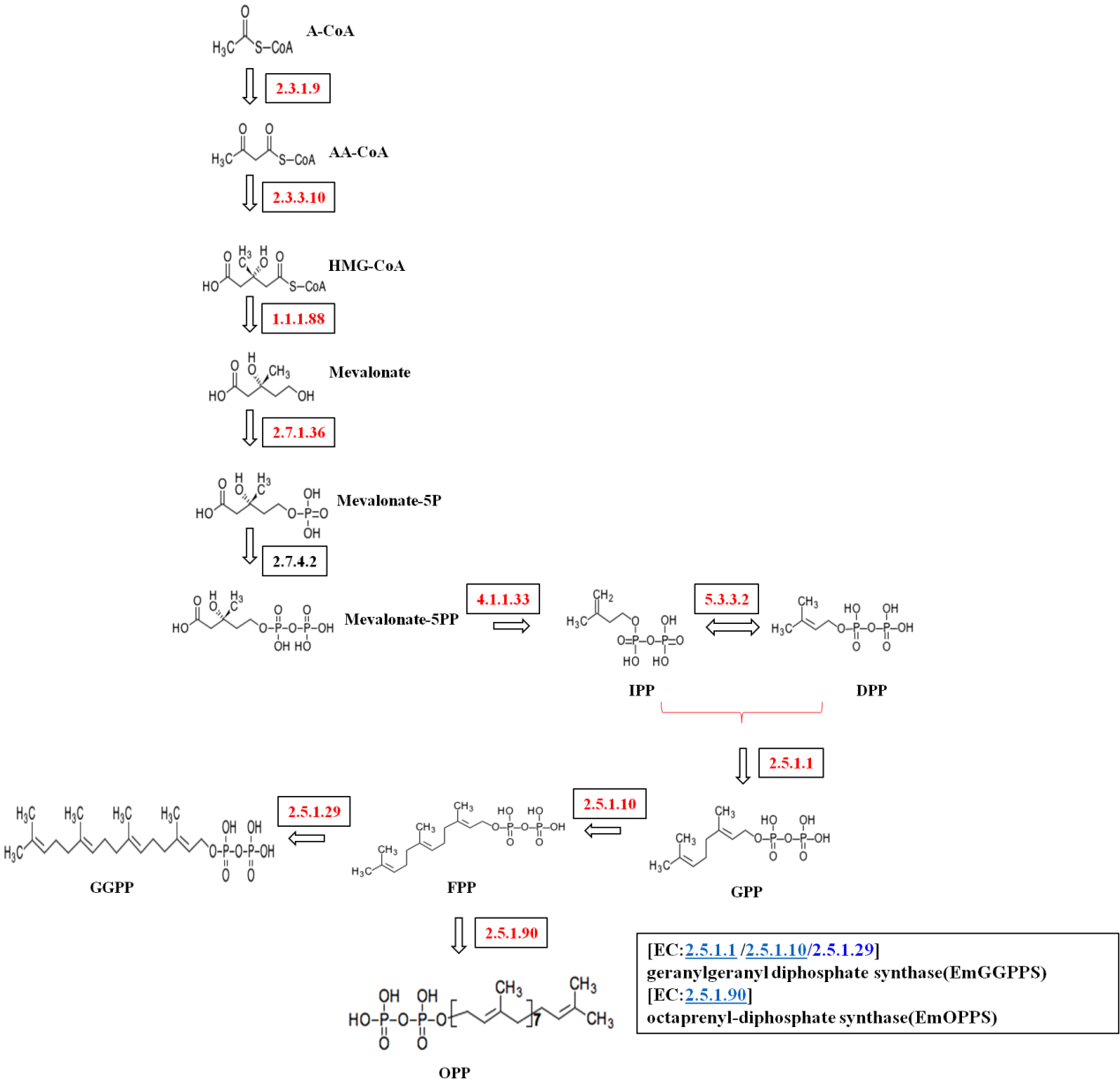
**

Figure S2. Chromatography of MK-n (n=4, 5, 6, 7, 8) by HPLC system. (A) The sample was extracted from monoculture system of *E. meningoseptica*, (B) The sample was extracted from coculture system of *E. meningoseptica and E. coli*.

**
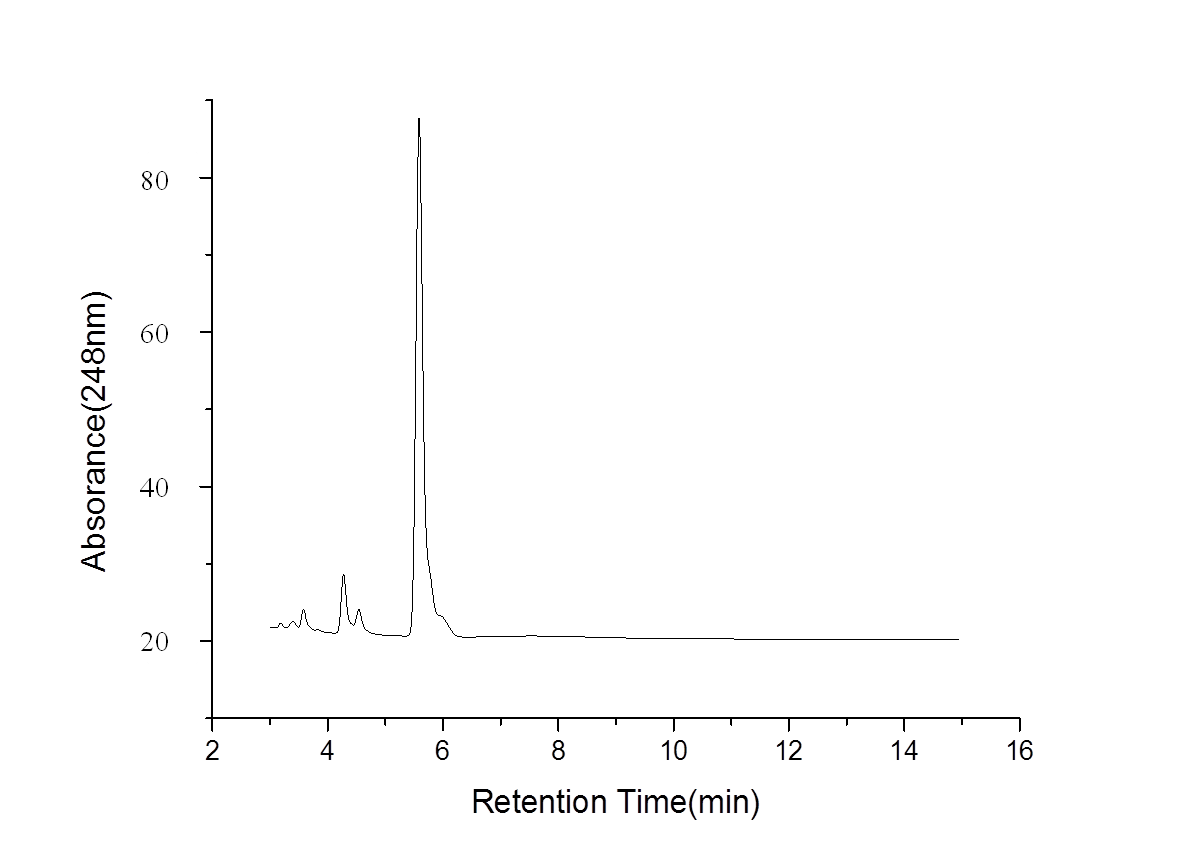
**

(A)

MK-4

MK-5

MK-6

**
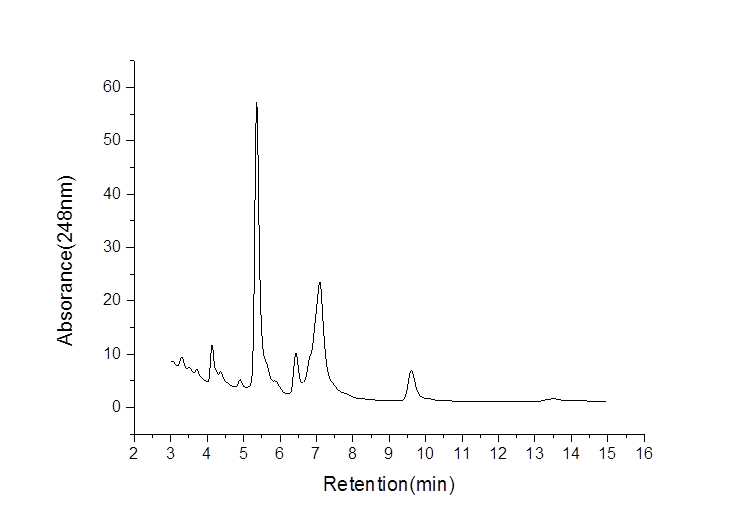
**

(B)

MK-4

MK-5

MK-8

MK-7

MK-6

Figure S3. (A) The purification of GB1-OPPS. M: protein molecular weight marker; lane 1:The total cell lysate supernatant flows through the column. Lanes 2–6: wash fractions with 10, 15, 20, 25, 30 mM imidazole, respectively. Lanes 7–8: Elution fractions with 250 mM imidazole, respectively. (B)Purified GB1-*Em*OPPS proteins was confirmed by mass spectrometry.


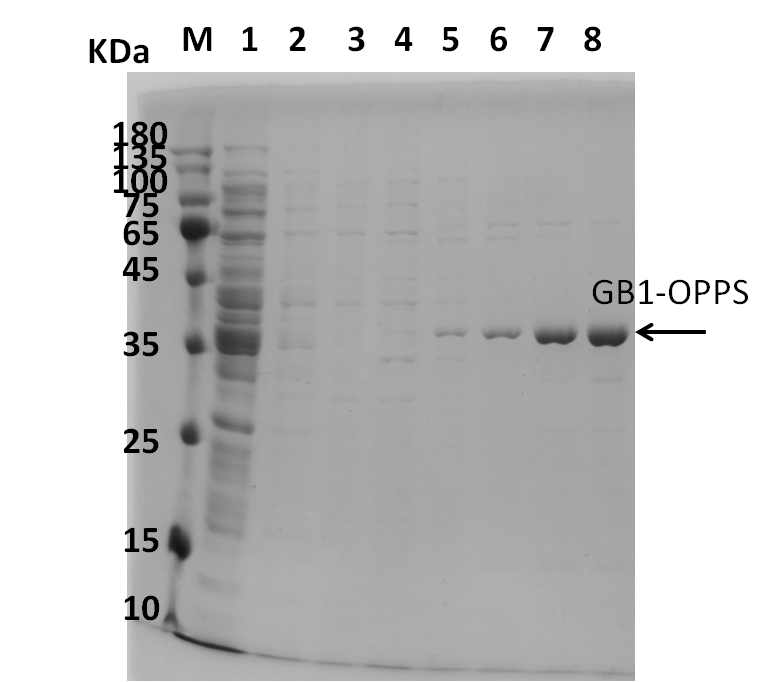


**(A)**

**(B)**

**
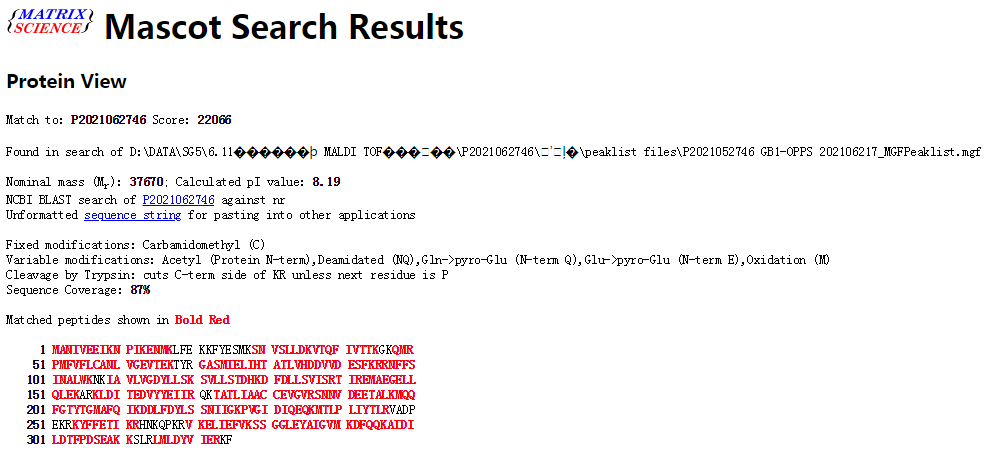
**

**Fig. S4.** Enzymatic assay of EmOPPS in vivo and vitro. (A, B) LC-MS analysis of the GB1-EmOPPS products with farnesyl diphosphate (FPP) and isopentenyl diphosphate (IPP) as substrates after hydrolyzation.. (C, D) J01 strains could accumulated MK-n (n=6, 7, 8). LC-MS analysis of the production of this strains. (A, C)Horizontal graphs refer to result of HPLC and vertical graphs (B, D) refer to mass spectra.


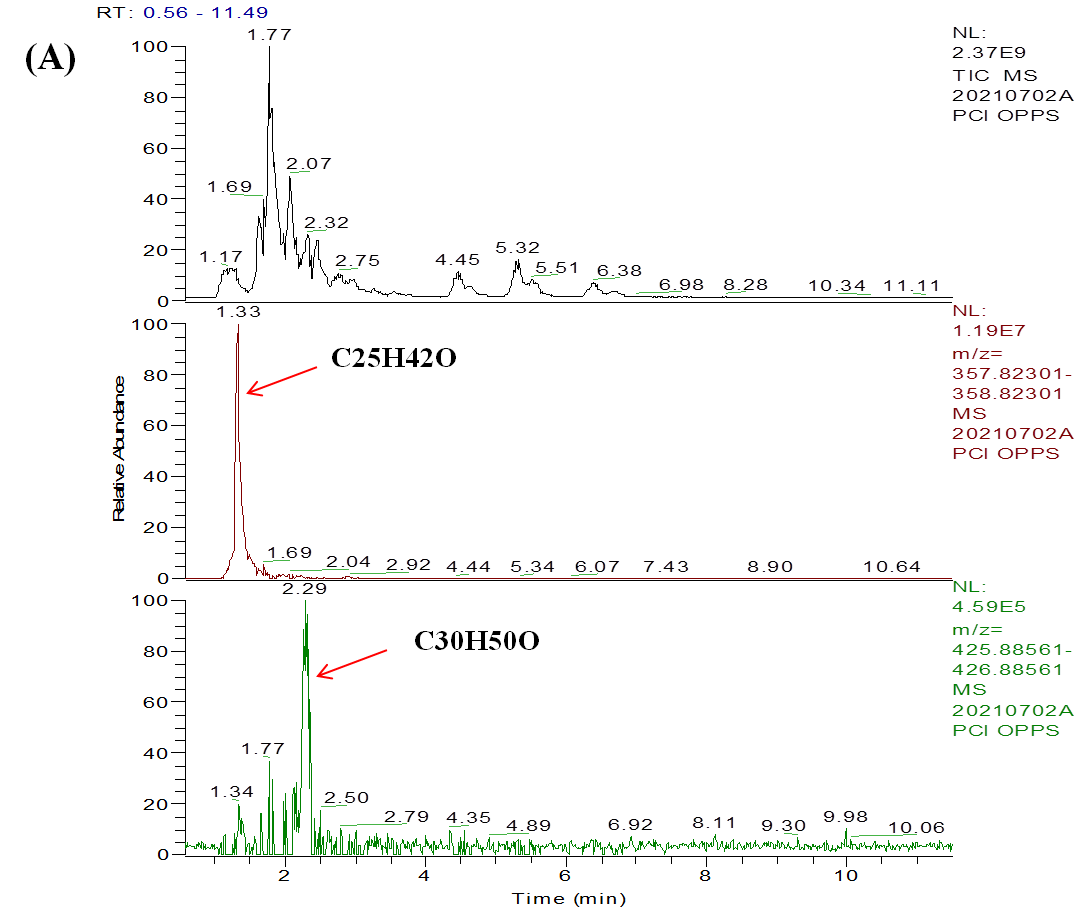


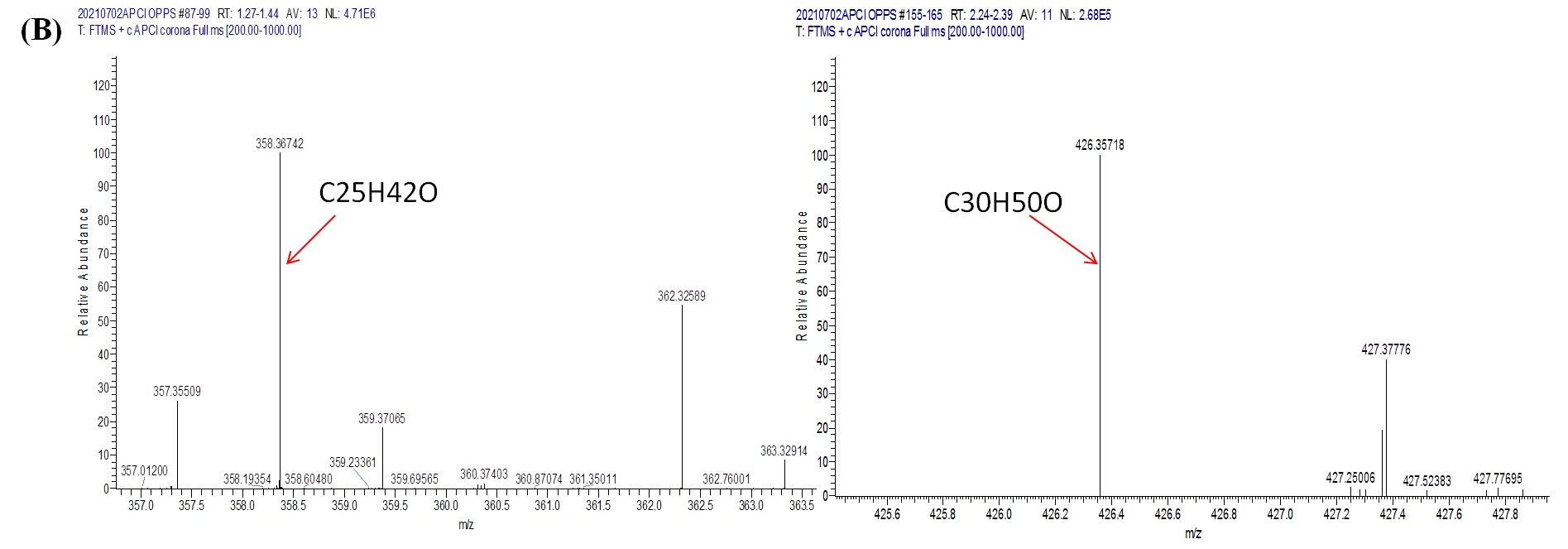


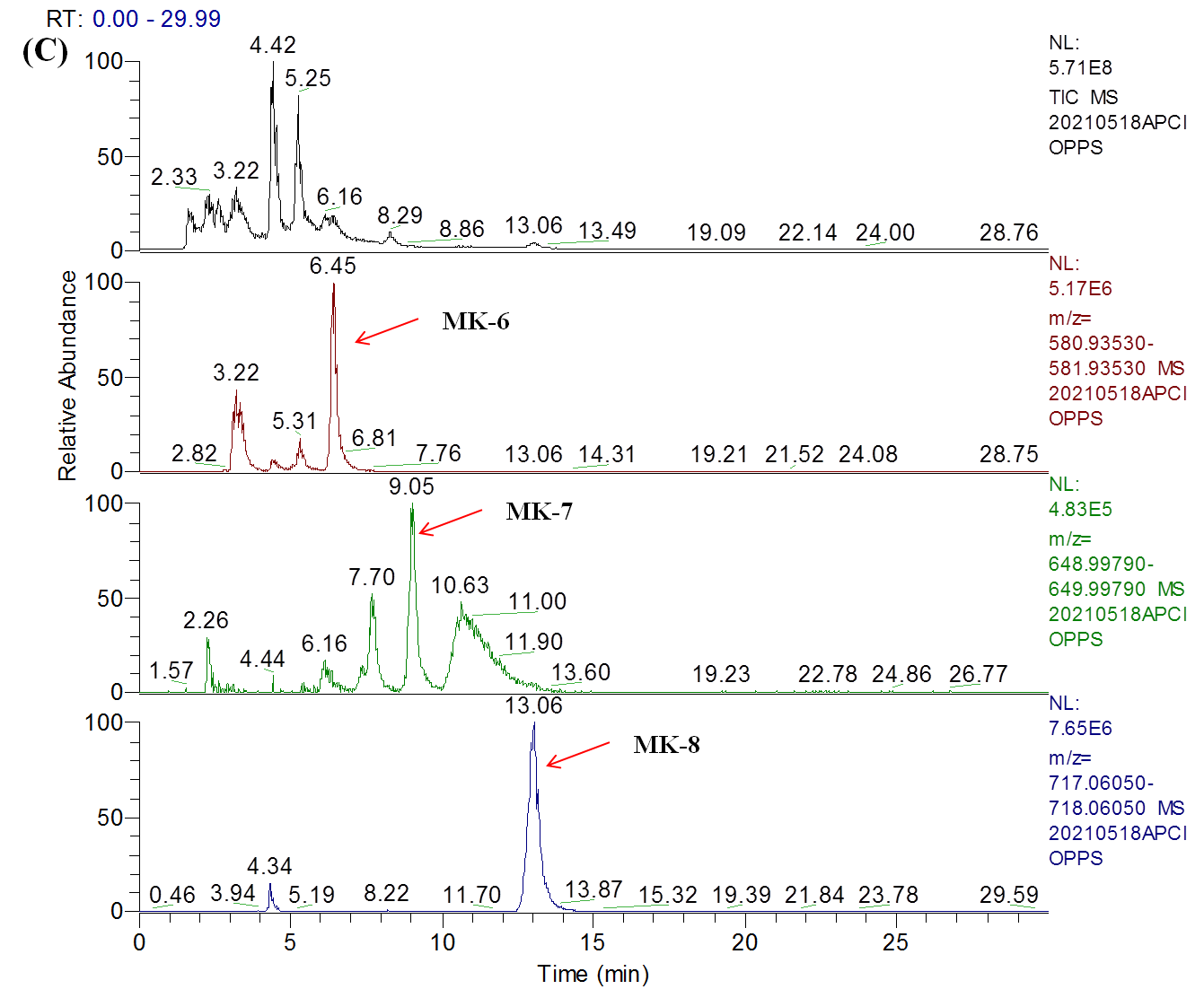


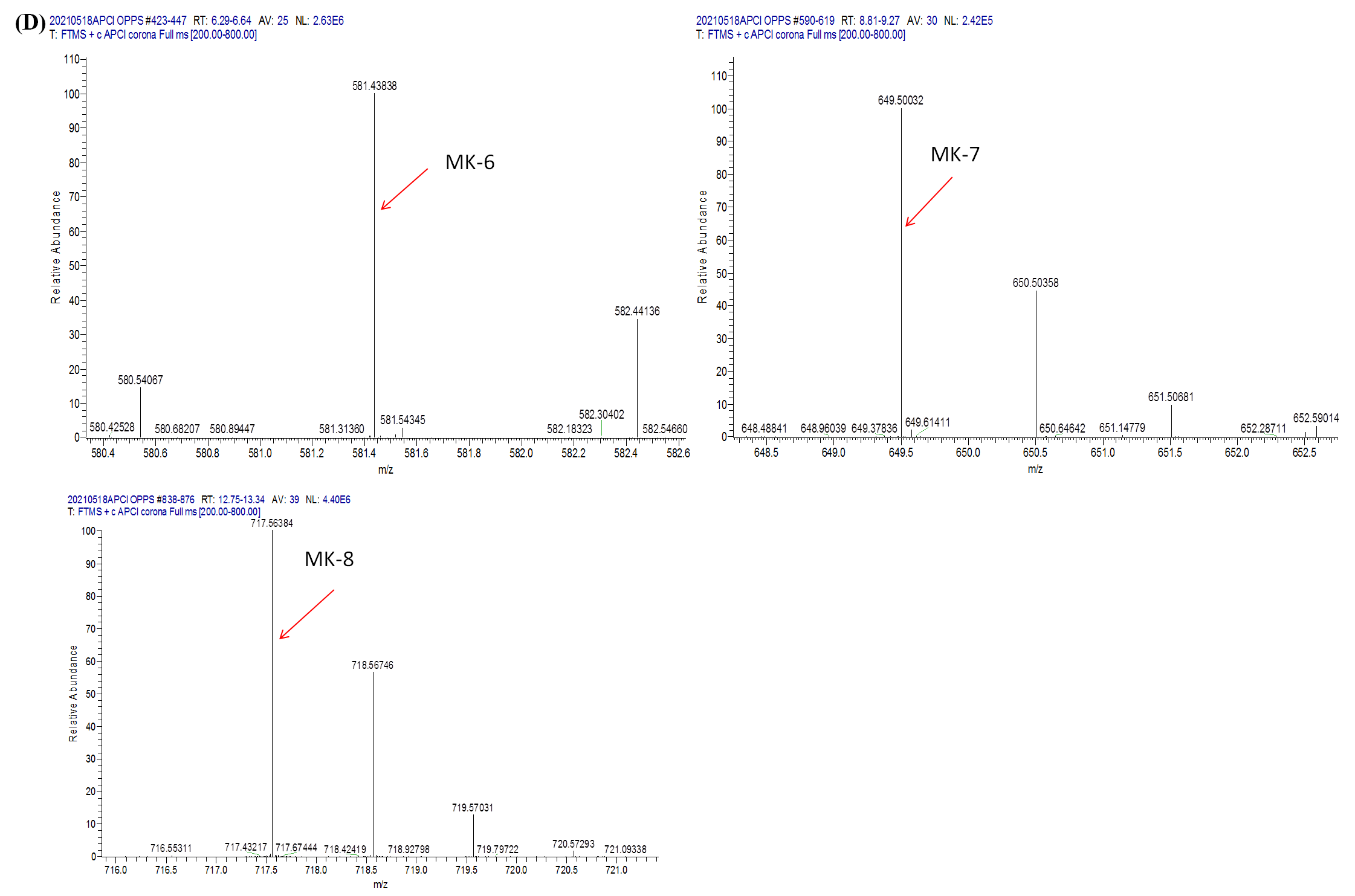


Figure S5. The comparison of the mass spectrum of the fermentation results of the J01, J02 strain with that of the MK-n (n = 4, 5, 6, 7) standard, (A) MK-4, MK-5, MK-6, and MK-7 standard, respectively, for MS analysis. (B)The mass spectrum results of monocultured *E. coli* producing MK-8 as a reference.


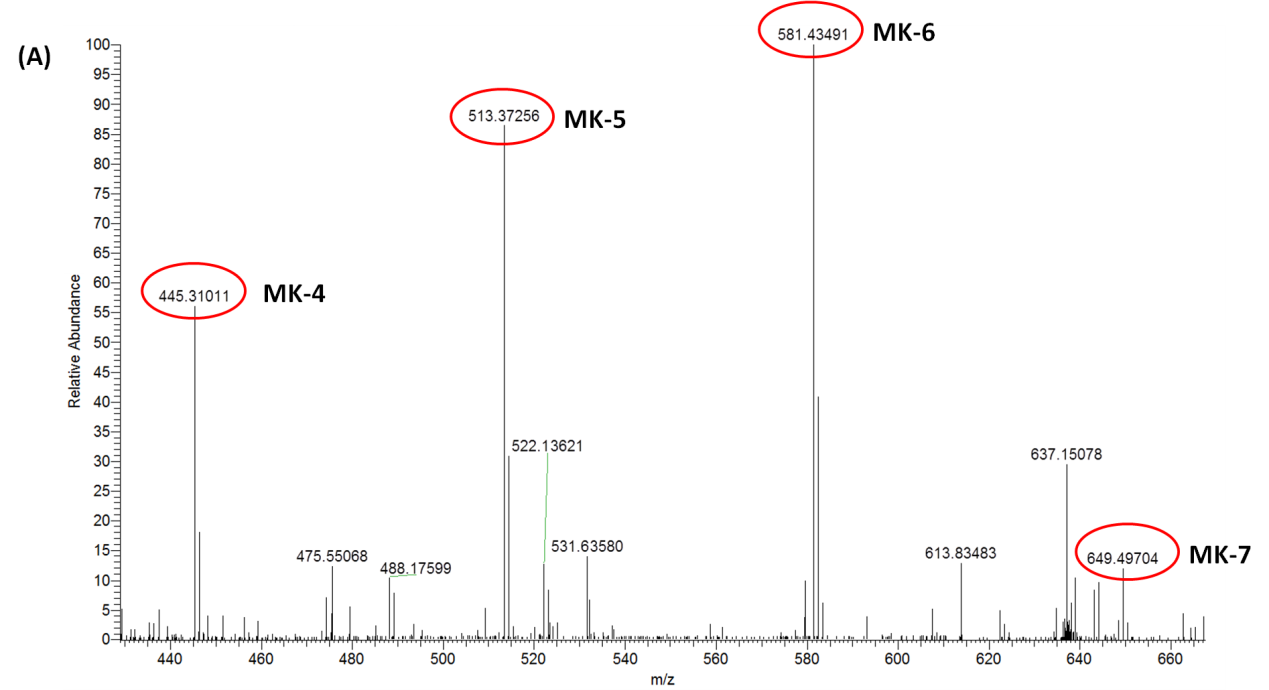


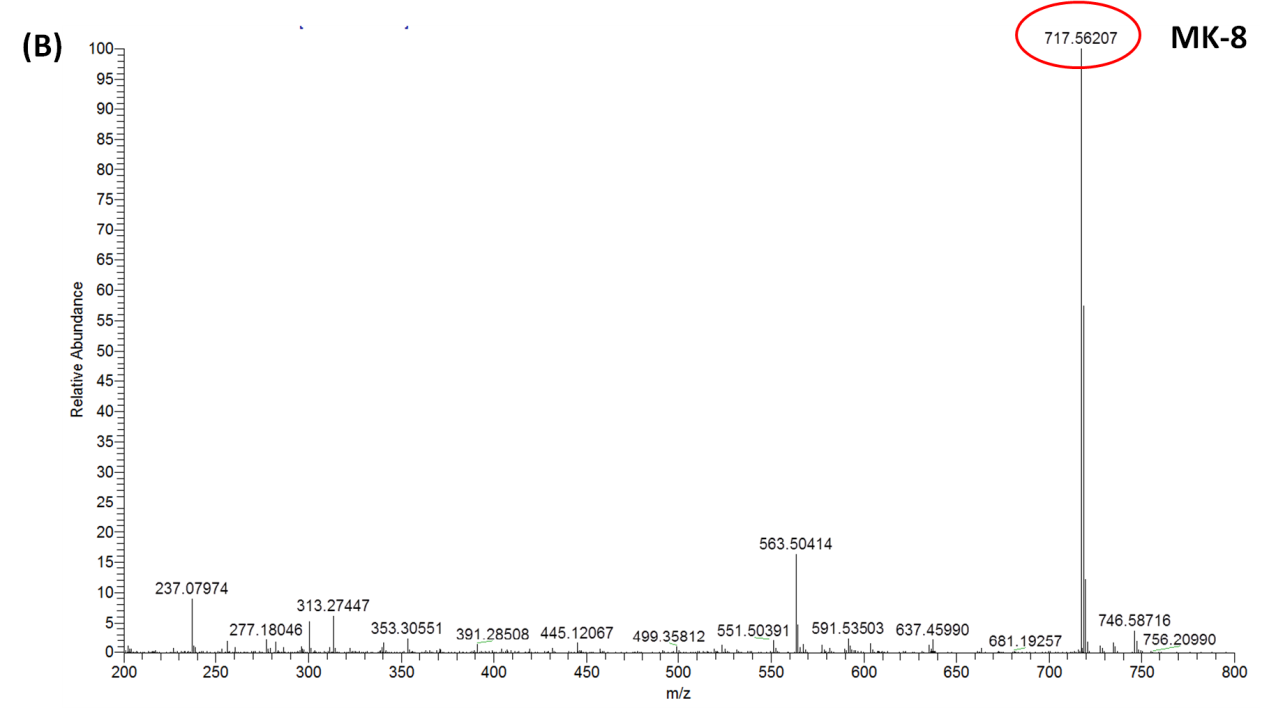


Figure S6. Analysis and identification of mevalonolactone by GC-MS from fermentation supernatant.


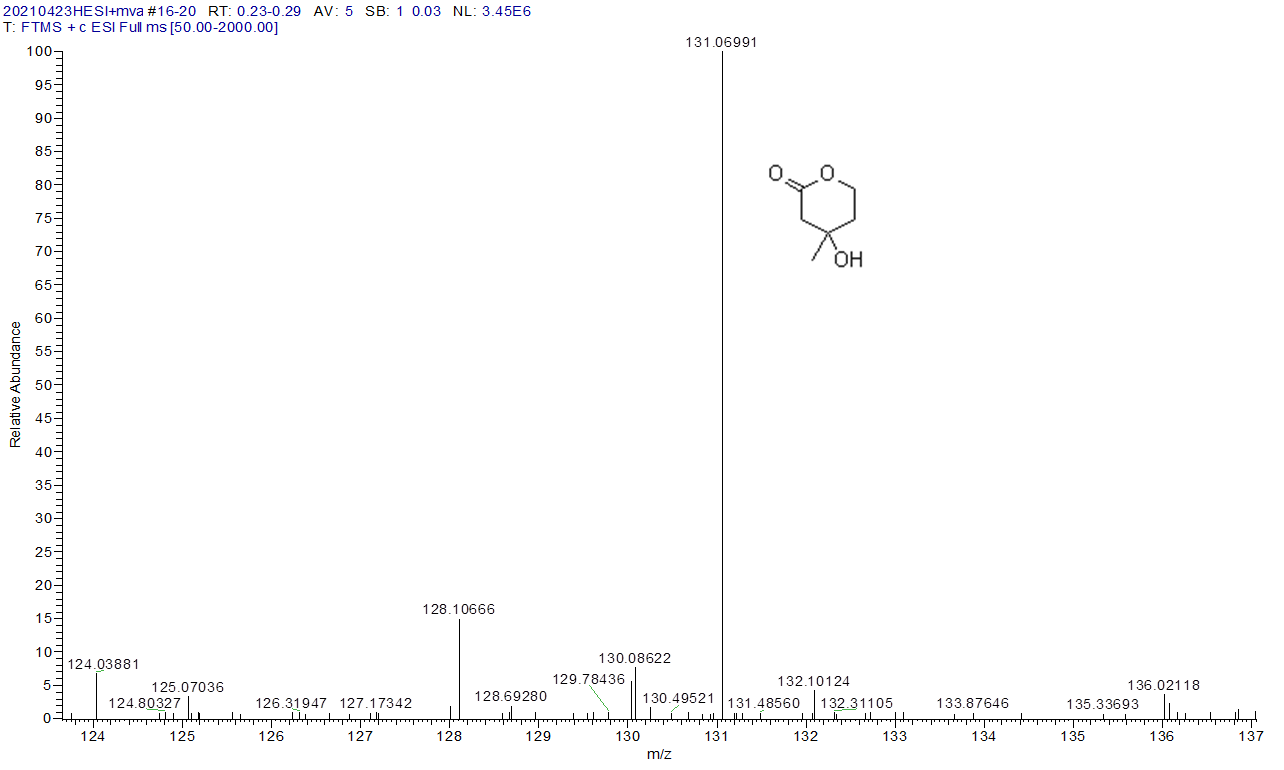


Figure S7. The monoculture of different *E. coli* strains as comparison. The error bars represent the standard error of at least three biological replicates. Statistical significance (*p* < 0.01) compared to the original strains.


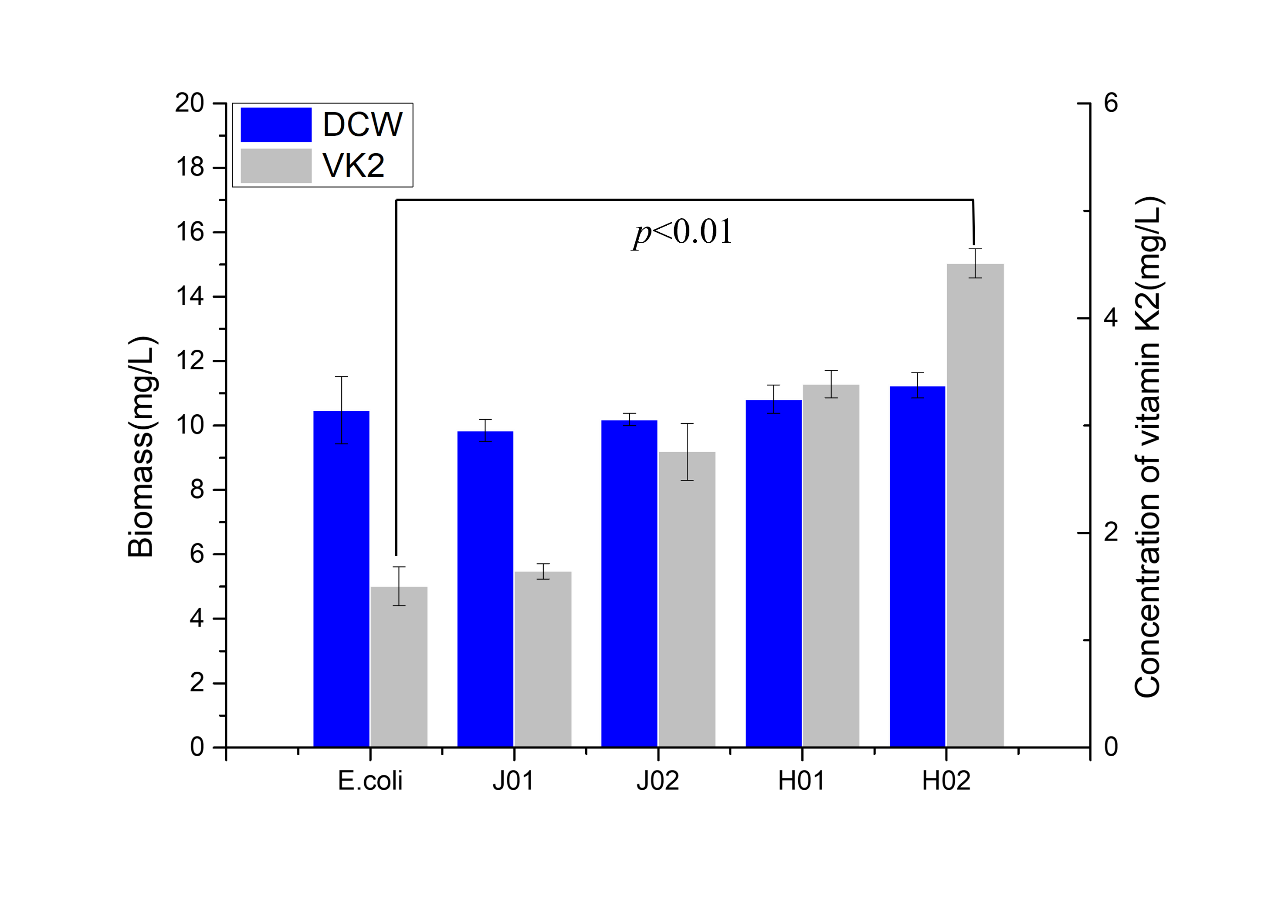


Figure S8. Visualization of different inocula by confocal laser scanning microscope. (A) *E. meningoseptica.* (B) *E. coli*. (C,D) *E. meningoseptica* and *E. coli* co-culture. Red circles surround the connected *E. meningoseptica* and *E. coli* cells in view. (C) Bright field; (D) Fluorescence image merged image.

Table S1 Masses and identities of LC/MS analyses results.

| **Medium** | Parent ion[M+H]^+^ | Exact mass (m/z) |
| --- | --- | --- |
| C25H42O^a^ | [C25H42O+H] | 358.36 |
| C30H50O^b^ | [C30H50O+H] | 426.35 |
| Menaquinones-4（MK-4） | [C31H40O2+H] | 445.31 |
| Menaquinones-5（MK-5） | [C36H48O2+H] | 513.37 |
| Menaquinones-6（MK-6） | [C41H56O2+H] | 581.43 |
| Menaquinones-7（MK-7） | [C46H64O2+H] | 649.50 |
| Menaquinones-8（MK-8） | [C51H72O2+H] | 717.56 |
| Mevalonolactone | [C6H10O3+H] | 131.06 |

Note:

a, (2E,6E,10E,14E)-3,7,11,15,19-Pentamethylicosane-2,6,10,14,18-pentaene-1-ol

b, Academic name unknown

Table S2 Oligonucleotide primers used in this study.

| Primer | Sequence (5′-3′) |
| --- | --- |
| EmHMGS-F GTTTAACTTTAATAAGGAGATATACCATGGATGAAAGTTGGAGTTGAAG  EmHMGS-R GCTGATGTTTCATATCTATATATTGGTAATATCTGGC  EmHMGR-F CAATATATAGATATGAAACATCAGCCTATAG  EmHMGR-R TGCAGGCGCGCCGAGCTCGAATTCGTTAATTTCCTCTCAGTTTTTCC  SceMK-F GGACAGGCTTTAAAAGCTTGATATCGAATTCC  SceMK-R CGCAGCAGCGGTTTCTTTACCAGACTCGAGTTATGAAGTCCATGGTAAATTC  ScePMK-F GGACAGGCTTTAAAAGCTTGATATCGAATTCC  ScePMK-R CGCAGCAGCGGTTTCTTTACCAGACTCGAGTTATGAAGTCCATGGTAAATTC  EmPVD-F GTTTAACTTTAAGAAGGAGATATACCATGGTGGAAAAACAATTTTTAGGAAATATAA  EmPVD-R ATCTGCCTCCTAAAATCTCATTTTATCTTTTACCAC  EmIDI-F AGGAGATATACATATGGCAGATCTCAATTGGGAAGAAAAAGTTGTACTTGTAACC  EmIDI-R GATATCAAGCTTTTAAAGCCTGTCCCGGTATTC  MBP-F GTTTAACTTTAAGAAGGAGATATACCATGGGCATGAAAGAACATCACCATCACC  MBP-R CAAAAAACTTCATAGAACCTTGGAAATAAAGATT  EmGGPPS-F TTCCAAGGTTCTATGAAGTTTTTTGATCAATATCAGGACC  EmGGPPS TTTCTGTTCGACTTAAGCATTATGCGGCCGCTTATGAATCTCTTTTTAATAAATAATTTGC  GB1-F GTTAAGTATAAGAAGGAGATATACATATGATGGACACTTACAAATTAATCC  GB1-R CAATATTAGCCATAGAACCTTGGAAATAAAGATTC  EmOPPS-F TTCCAAGGTTCTATGGCTAATATTGTAGAGGAAATCAAAAA  EmOPPS-R GTTTCTTTACCAGACTCGAGGGTACCCTTAGAATTTACGCTCGATGAC  EcMenA-F AATAGGGATCCGAATTCGAGCTCCGTCGACTCGCGTTCAATCATTTTCATCATTG  EcMenA-R TATTCGTTGTCATGAGTATTACCTCCTATGCTGCCCACTGGCTTAG  EmMenE-F CAGTGGGCAGCATAGGAGGTAATACTCATGACAACGAATAAAAATAATATTT  EmMenE-R ATCTCAGTGGTGGTGGTGGTGGTGCTCGAGTTACTTTGTTGCTTTATAAATAGTTG | |
|  | |
